# Integration of proteomic and genomic approaches to dissect seed germination vigor in *Brassica napus* seeds differing in oil content

**DOI:** 10.1186/s12870-018-1624-7

**Published:** 2019-01-11

**Authors:** Jianwei Gu, Dalin Hou, Yonghong Li, Hongbo Chao, Kai Zhang, Hao Wang, Jun Xiang, Nadia Raboanatahiry, Baoshan Wang, Maoteng Li

**Affiliations:** 1 Department of Biotechnology, College of Life Science and Technology, Huazhong University of Science and Technology, Wuhan, China; 2 Hubei Key Laboratory of Economic Forest Germplasm Improvement and Resources Comprehensive Utilization, Hubei Collaborative Innovation Center for the Characteristic Resources Exploitation of Dabie Mountains, Huanggang Normal University, Huanggang, China; 3 Hybrid Rapeseed Research Center of Shaanxi Province, Shaanxi Rapeseed Branch of National Centre for Oil Crops Genetic Improvement, Yangling, China; 4 Hubei Institute of New Socialist Countryside Development, Hubei Engineering University, Xiaogan, China; 5 College of Life Science, Shandong Normal University, Jinan, China

**Keywords:** Brassica, Seed germination vigor, 2D-DIGE, QTL analysis

## Abstract

**Background:**

Rapeseed (*Brassica napus, B. napus*) is an important oil seed crop in the world. Previous studies showed that seed germination vigor might be correlated with seed oil content in *B. napus*, but the regulation mechanism for seed germination has not yet been explained clearly. Dissecting the regulation mechanism of seed germination and germination vigor is necessary.

**Results:**

Here, proteomic and genomic approaches were used to analyze the germination process in *B. napus* seeds with different oil content. The identification of 165 differentially expressed proteins (DEPs) in the germinating seeds of *B. napus* with high and low oil content was accomplished by two-dimensional fluorescence difference in gel electrophoresis (2D-DIGE). The comparative proteomic results revealed that seeds with high oil content had higher metabolic activity, especially for sulfur amino acid metabolism. Thirty-one unique genes were shown to be significantly changed during germination between the seeds with high and low oil content, and thirteen of these genes were located within the confidence interval of germination-related quantitative trait locus (QTLs), which might play an important role in regulating seed germination vigor.

**Conclusions:**

The present results are of importance for the understanding of the regulation mechanism for seed germination vigor in *B. napus*.

**Electronic supplementary material:**

The online version of this article (10.1186/s12870-018-1624-7) contains supplementary material, which is available to authorized users.

## Background

Seed germination is a critical development process in the life cycle of spermatophyte. Successful seed germination and normal seedling construction are the decisive factors for plant reproduction [1, 2]. Thus, research on the regulation mechanism of seed germination is important for agricultural production.

*Brassica napus* is the second most important oilseed crop in the world, which occupies approximately 13–16% of the world vegetable oil production [3]. The seed germination and germination vigor are affected by many factors in *B. napus*, such as salt [4, 5], temperature [6, 7], plant hormones [8], and aging [9]. The seed germination vigor of rapeseed could be enhanced by priming [10, 11]. In addition, a genome-wide association analysis showed that seed germination and radicle growth were strongly environment-dependent in *B. napus* [12]. Conditions during seed production and storage have a profound effect on seed vigor, and the performance of seed dormancy was related to differences in germination performance [12]. A number of genes, proteins or QTLs that correlated with germination were identified [12], and these genes included *SNOWY COTYLEDON 1* (*Bna.SCO1*), *ARABIDOPSIS TWO-COMPONENT RESPONSE REGULATOR* (*Bna.ARR4*) and *ARGINYL-t-RNA PROTEIN TRANSFERASE 1* (*Bna.ATE1*). The homologous genes in *Arabidopsis* have been shown to participate in seed germination and seedling growth [13–15]. A 90 kD heat shock protein (HSP 90) was detected in the elongating axes and cotyledons of germinating seedlings and could be induced by stress in *B. napus*, which highlights a role for HSP 90 during seed germination [16]. Jian et al. identified 19 QTLs that are associated with seed germination rates under salt and drought stress in *B. napus* [17].

Seed germination vigor refers to the potential to accomplish rapid, precise germination and seedling formation from seeds in field conditions [18]. Seed vigor has been shown to be closely related to crop field performance in variety of crops, especially in *B. napus* [18–21]. After long-term storage, the seeds gradually deteriorate and lose their activity, which is known as the aging of seeds. High temperature and high humidity storage conditions could accelerate the aging process of seeds [9, 22]. This is an important problem in practical seed production, and a significant cost has been paid in order to reduce the loss of seed vigor during storage [23, 24]. Based on the present results in *Arabidopsis*, oxidative stress was an important factor in seed aging [25]. The loss of seed vigor during aging could be attributed to two aspects: one was protein changes in dry seeds, and the second was that low-activity seeds cannot express normal proteomes during germination [25]. The key mechanisms for maintaining seed vigor include the translation capacity, seed storage mobilization and antioxidant efficiency [25]. Additionally, some late embryogenesis abundant (LEAs) proteins and HSPs also play an important role in maintaining seed vigor [25].

The fatty acid (FA) composition of rapeseed was changed dramatically in the aging process. In addition, the aging of seeds could also prolong the germination time, reduce the germination and the overwinter rate, and decrease the rapeseed yield [21, 26]. *B. napus* seeds are always sown in autumn and harvested in late spring, so the seeds will go through a hot and humid summer, which can lead to the loss of germination vigor. Therefore, a comprehensive understanding of the molecular mechanism of seed germination and germination vigor is important for reducing the loss of seed vigor in *B. napus*. Yin et al. demonstrated that the primary factor contributing to the aging of *B. napus* seeds might not be the accumulation of reactive oxygen species (ROS) but the accumulation of endogenous abscisic acid (ABA) during seed aging [27]. Morris et al. have found that two genes, *BoLCVIG1* and *BoLCVIG2,* and a QTL (Reduced ABscisic Acid 1, RABA1) were associated with the determination of seed vigor in *Brassica oleracea*, and these genes were associated with ABA metabolism or seed sensitivity to ABA [28]. In addition, proteins that are involved in metabolic and protein transport were reduced by artificial aging treatment, whereas actin, mannose-binding lectin superfamily protein, glycosyltransferase, beta-glucosidase and other cell and cell wall structure-related protein content increased [27]. Moreover, previous studies also have shown that the loss of seed vigor during storage in *B. napus* seeds with different oil content is distinct [9]. Similarly, the loss of seed vigor in low oil-containing seeds was significantly lower than that of seeds with high oil content under the same conditions [26]. These results indicate that the seed vigor of rapeseed might be correlated with oil content.

The advent of powerful statistical methods for linkage-based QTLs mapping combined with the available diversity of the germplasm and the completion of reference genome sequencing in *B. napus*, allowing the genetic architecture of complex traits to be uncovered in *B. napus* [29–32]. The combination of QTL mapping and multi-omics studies provides a powerful framework for the functional characterization of genes [33]. For instance, this strategy has been used to identify genes controlling lipid biosynthesis in maize [33]. In this class of approaches, the candidate genes can be narrowed by utilizing multiple datasets [33]. These results indicate that the identification of candidate genes could be easier and more reliable by combining ‘-omics’ methods and QTL mapping.

The decreased quality and germination vigor of *B. napus* seeds during long-term storage is a substantial problem. Therefore, improving seed germination vigor and weakening or eliminating the adverse effects of aging on seed germination vigor are related to the improvement of the yield and the quality of the domestic edible oil, which has great significance. This requires a detailed understanding of the regulation mechanism for seed germination and germination vigor in *B. napus*. In this paper, DEPs were observed in the seed germination process of rapeseed lines with high and low oil content, and important candidate genes were identified by combining the QTL analysis for controlling of seed germination vigor in *B. napus*. The results lay the foundation for the ultimate improvement of seed germination vigor and weakening or eliminating the adverse effects of aging on seed germination in *B. napus*.

## Results

### Effects of oil content on seed germination vigor in *B. napus*

**Fig. 1:**
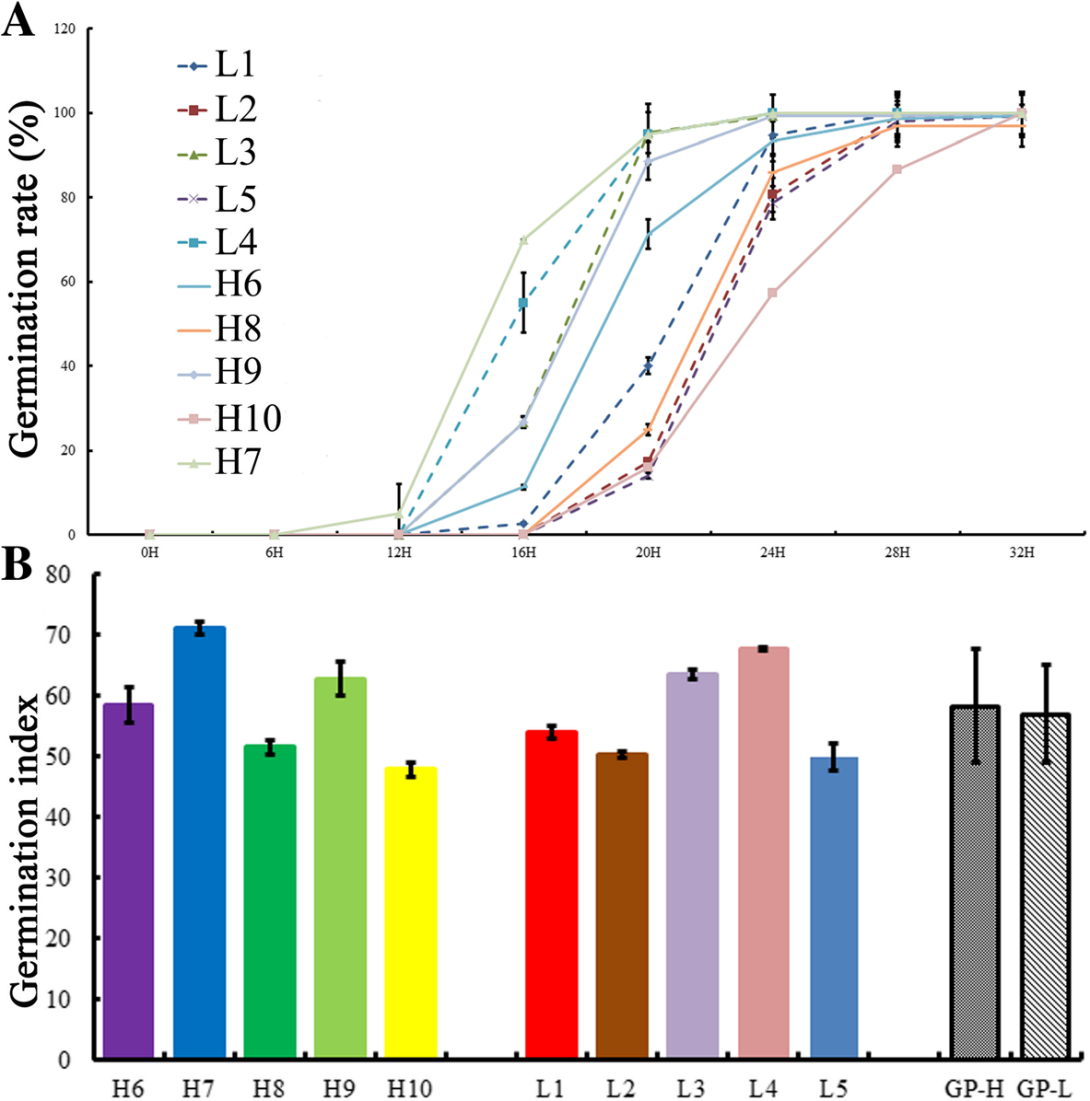
Comparison of germination in *B. napus* with different oil content. **a**, the germination rates of seeds with different oil content. **b**, the germination index of seeds with different oil content. H6: 14QT124 (55.59%); H7: 12WH191 (55.83%); H8: 14QT142 (56.56%); H9: 14QT026 (57.01%); H10: 14QT129 (57.33%); L1: 14QT078 (33.27%); L2: 14QT050 (36.65%); L3: 14QT170 (39.33%); L4: Ken C8 (40.06%); L5: 14QT001 (41.18%). GI-H and GI-L represent the average germination index of the high oil and low oil groups, respectively

To verify whether the oil content could affect the seed germination vigor, 10 cultivars with high oil content and 10 with low oil content were used for the germination experiment. It was revealed that some seeds began to germinate at 12 h after imbibition (HAI), and most had completed germinating at 32 HAI. Further analysis showed that most of cultivars with high oil content could initiate germination more quickly than those with low oil content (Additional file 1: Table S1, Fig. 1a and Additional file 2: A), and also that the cultivars with high oil content had a relatively high germination index (GI) (Fig. 1b and Additional file 2: B). ANOVA analysis was performed to compare the GIs of high and low oil-content materials (Additional file 1: Table S1). The result presented that the difference in the GI between the two groups was indeed not significant, but the average GI of high oil content materials is clearly higher than that of low oil content materials. This result indicates that the oil content could affect the germination vigor of *B. napus* seeds.

### Major metabolite changes in germinating seeds with high and low oil content

**Fig. 2:**
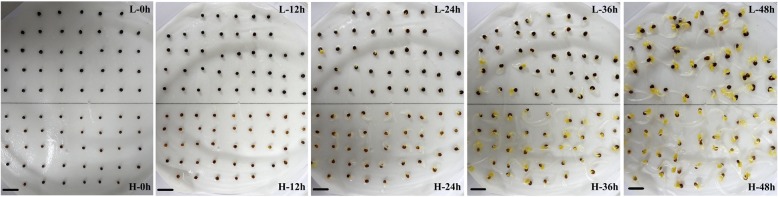
Comparison of seed germination process between 12WH191 (H) and KenC8 (L). Compared with low oil-content seed, high oil-content seeds showed faster germination and post-germination growth. The seeds were imbibed in water at 28 °C under dark. Photographs were taken at 0, 12, 24, 36 and 48 HAI. Scale bar represents 1 cm

To better understand the differences in seed germination vigor between the seeds with high and low oil content, two cultivars, KenC8 and 12WH191, with oil contents of 40.06 and 55.83% and distinct germination vigor levels, were selected for further analysis. The germination of 12WH191 was much faster than that of KenC8, and the radicle broke through the seed coat as early as 12 HAI in some 12WH191 seeds. In two seeds that germinated after 24 HAI, the radicle protruded from the seed coat and hypocotyl elongation occurred (Fig. 2). After 48 HAI, two examples had started seedling development: the radicle developed into young roots and root hair grew vigorously, cotyledons began to stretch, and the seed coat began to fall off the cotyledons (Fig. 2). The moisture of the seeds changed significantly during germination, but there were no significant differences in the trend between the two cultivars (Additional file 3). The water content of the seeds increased rapidly to approximately 40% in the first 12 h of germination and then gradually increased to approximately 65% at 36 HAI. Finally, at 48 HAI, the water content increased rapidly to approximately 80%.

To evaluate the mobilization of storage reserves in seeds during germination and post-germination, the total oil content was measured (Additional file 3), and it was revealed that the changing tendency of oil content in germinating KenC8 and 12WH191 seeds was similar, and significant changes only occurred during post-germination seedling growth (Additional file 3). This result suggests that the storage of lipids might be mobilized during germination and are mainly consumed during the post-germination stages. The difference in the relative content of FAs was dramatically observed in these two materials (Additional file 4). In dry seeds, the content of C18:3 in KenC8 was significantly lower than that in 12WH191; otherwise, the content of C18:1 and C18:2 in KenC8 were higher than that in 12WH19. Though some changes of C18:3, C22:1, eicosanoids (C20:0), eicosanoic acid (C20:1) and palmitic acid (C16:0) could be observed in Ken C8 and 12WH191 during germination, the FAs remained relatively stable during germination and post-germination growth.

The changes in protein and sugar content were observed during the germination process (Additional file 3). It was revealed that the total protein content of both KenC8 and 12WH191 increased rapidly after imbibition (0–12 HAI) and was followed by a gradual decrease. At 72 h, the total protein content of both had been reduced to approximately 30 mg/g. This result suggests that in the early stages of germination (0–12 h), there is a large amount of new protein synthesis in the germinating seed, and a large amount of stored protein is consumed as the source of energy and substance metabolism for seed germination afterwards. In the early stages of germination, the total sugar content in the seeds decreased gradually. The total sugar content of both cultivars increased rapidly following 36 HAI, suggesting that the sugars in the seeds might be the source of energy and substance metabolism required for early seed germination.

Other physiological characteristics that might reflect the physical and metabolic energy use during germination were also determined, including soluble sugar, pyruvate and acetyl-CoA (Additional file 3). There were no significant differences in the pyruvate content between KenC8 and 12WH191 during germination. In dry seeds, the soluble sugar content in 12WH191 was essentially equal to Ken C8. However, the content of soluble sugar in KenC8 increased rapidly during early germination (0–12 h) and then decreased gradually, and the soluble sugar content in 12WH191 was always lower than that in KenC8 during germination. The change of acetyl-CoA was nearly same in KenC8 and 12WH191, which gradually decreased in the early stage of germination, and then gradually increased, but began to decrease again after 36 HAI. The content of acetyl-CoA in KenC8 was always lower than that in 12WH191, especially at 0–24 HAI. The content of acetyl-CoA in 12WH191 was 2–3 times higher than that in KenC8. This might imply that the substance and metabolic energy usage in low oil-containing seeds was significantly lower than that in high oil-containing seeds.

As an important plant hormone, the content of ABA was also compared between 12WH191 and KenC8 during germination (Additional file 3). It was shown that the content of ABA decreased along with germination in both cultivars, but it was significant higher in KenC8 seeds with lower oil content. This result implies that ABA might participate in the determination of seed vigor in *B. napus* seeds with different oil content.

### Microstructure comparison of embryonic cells during germination in seeds with high and low oil content

**Fig. 3:**
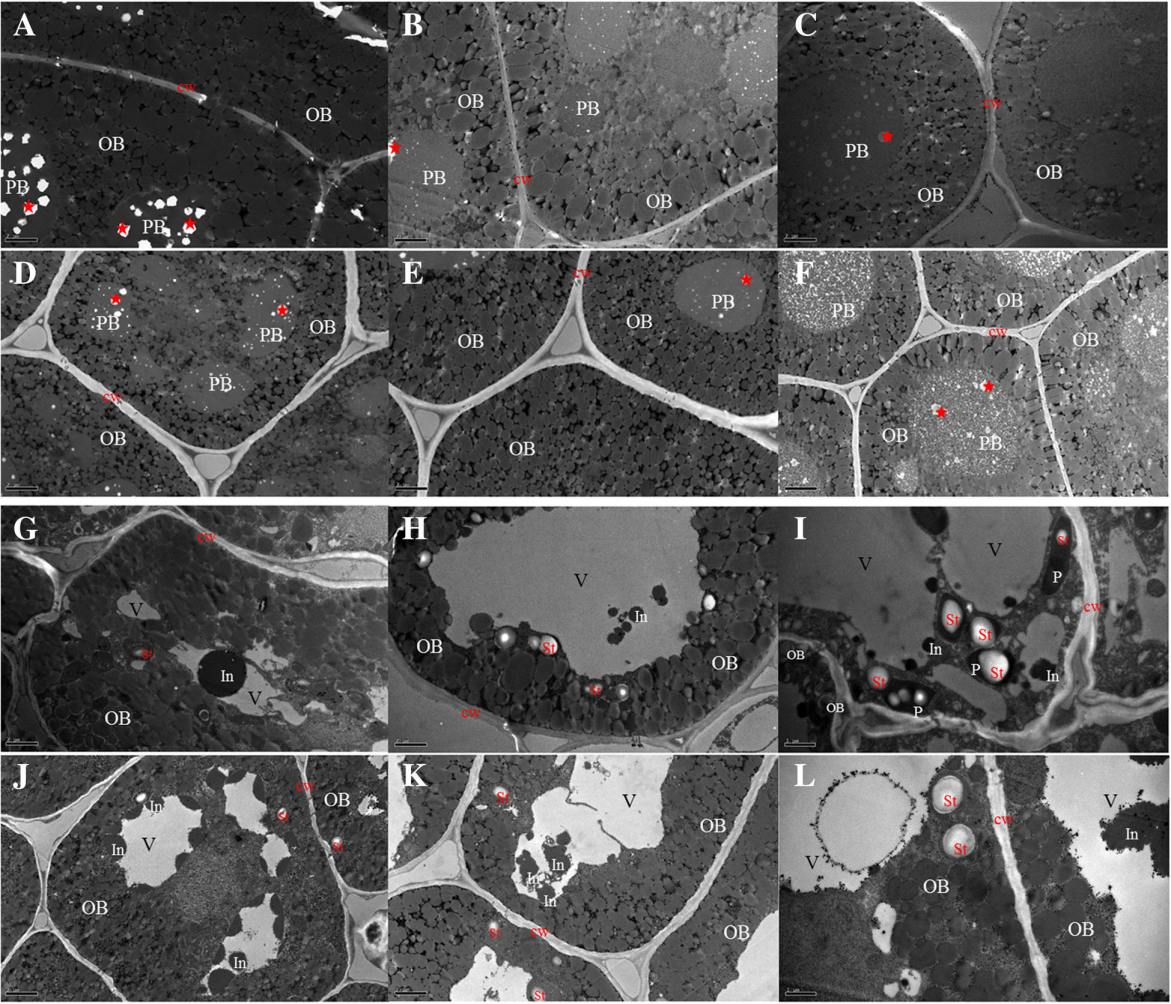
Comparison of the high and low oil-containing *B. napus* cotyledon cells during in vitro germination and seedling growth. **a**-**c** and G-I: the development of cotyledon cells in high oil-content material (12WH191) imbibed in water at 28 °C under dark. **d**-**f** and **j**-**l**: the development of cotyledon cells in low oil-content material (KenC8) imbibed in water at 28 °C under dark. Significant difference can be seen in the mobilization of PBs and OBs and the accumulation of starch. **a** and **c**, dry mature seeds; **b** and **d**, 12 h of in vitro germination; **g** and **j**, 2 days of in vitro germination; **h** and **k**, 3 days of germination; **i** and **l**, 4 days of germination. PB: protein body; OB: oil body; V: vacuole; St: starch grains; P: plastids; cw: cell wall. Scale bar (**a**-**e**; **g**-**k**) =2 μm. Bar (**f** and **l**) =1 μm

The microstructural changes in cotyledons during germination were analyzed (Fig. 3), and it was revealed that most cells were filled with oilbodies (OBs) and protein bodies (PBs). In dry seeds, the cotyledon cells of 12WH191 had a smaller the interspace between OBs and thinner cell walls than those of KenC8. Electron-light and small rounded areas were present in the PB matrix (red stars) of 12WH191 and KenC8, but the volume of these inclusions in 12WH191 was relatively larger than those of KenC8 (Fig. 3 a and d). No significant changes were observed in 12WH191 and KenC8 after 12 HAI except that the volume and the numbers of inclusions in PBs were reduced (Fig. 3 b and e). After 24 HAI, significant changes were still not observed in 12WH191, but in KenC8, the electron density of PBs was significantly reduced and became similar to a fibrous granularity (Fig. 3 c and f). This suggests that the storage proteins in seeds of KenC8 had been mobilized and consumed at this stage. After 2 days of germination, the PBs in cotyledon cells of 12WH191 and KenC8 both became a vacuole structure and some of the electron-dense inclusions could be observed in these vacuoles (Fig. 3 g and j). These vesicular-like structures had fused after 3 days of germination and became a large vacuole located in the center of the cell, and the inclusions could still be observed in the vacuole. In addition, some starch grains began to appear in the cells, but cotyledon cells in 12WH191 apparently accumulated more starch grains (Fig. 3 h and k). After 96 h of germination, small inclusions were still present in the vacuole. Moreover, nearly no OBs were retained in the cotyledon cells of 12WH191, while some residual OBs could still be seen in KenC8. At the same time, it was observed that many plastids containing starch granules appeared in the cotyledon cell cytoplasm of 12WH191 and KenC8, and 12WH191 continued to accumulate more starch grains.

### Comparative proteomic analysis in seeds with high and low oil content

**Fig. 4:**
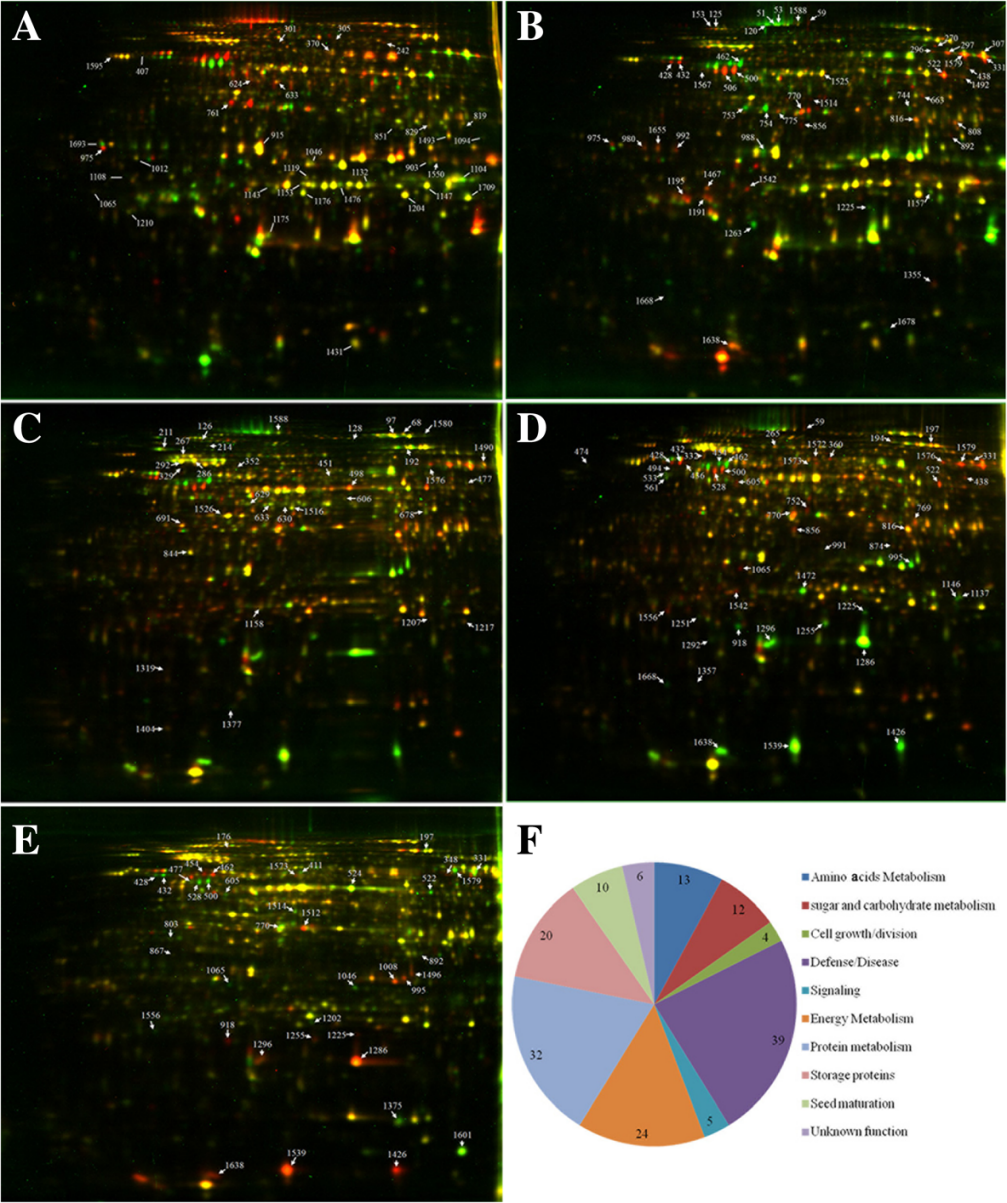
The 2D-DIGE maps and the functional categorization of DEPs in germinating *B. napus* seeds with high and low oil content. **a**-**e** represent the 2D-DIGE maps of 12WH191 (H) and KenC8 (L) germinating seeds at 0, 12, 24, 36 and 48 HAI. **f** represents the functional categorization of DEPs. Arrows show the protein spots that were highly expressed in low oil-containing seeds; lines show the protein spots that were highly expressed in high oil-containing seeds

Proteomic analysis of germinating seeds with high and low oil content in *B. napus* was conducted using 2D-DIGE. Approximately 1300 protein spots could be detected on each gel (Fig. 4 a-e). Based on the dynamic proteome at different germination stages, 165 DEPs were identified between the seeds with high and low oil content (Additional file 1: Tables S2 and S3). These DEPs were divided into 10 functional categories, including defense/disease (23.64%), protein metabolism (19.39%), energy metabolism (7.28%), sugar and carbohydrate metabolism (7.27%), seed mature protein (6.06%), signal pathway-related (3.03%), cell growth/division (14.55%), storage protein (12.12%), amino acid metabolism 2.42%) and proteins of unknown function (3.64%) (Fig. 4f and Additional file 1: Table S3). These DEPs corresponded to 114 specific *Arabidopsis* homologous genes (Additional file 1: Table S4). Among them, 28 homologous genes corresponded to multiple DEPs that involved stress and defense, protein metabolism, energy metabolism, sugar and carbohydrate metabolism, storage proteins, seed maturation and signal pathways. These diverse protein forms might be derived from variable splicing, post-transcriptional modifications and protein degradation metabolism. Moreover, we compared the genes corresponding to DEPs against to genes annotated as seed germination and seed specific from NCBI database. The result showed that only 9 and 4 genes corresponding to DEPs were annotated as seed germination and seed specific in NCBI, respectively (Additional file 1: Table S4).

Thirty-nine DEPs were involved in stress and defense, of which 22 spots were expressed significant higher in 12WH191 than in KenC8 in at least one germination stage. For example, compared to expression in KenC8, myrosinase-binding protein 2 (spot 59) was significantly higher in 12WH191 at 12 and 36 HAI (*P* < 0.05), while two other protein spots were higher in 12WH191 at 24 HAI (*P* < 0.05; Additional file 1: Table S3). Similarly, mannose-binding lectin superfamily protein (spot 522 and 1492), monodehydroascorbate reductase 1 (spot 1176), probable aldo-keto reductase 6 (spot 856) and dehydrin Rab18 (spot 1175) were highly expressed at one or more germination stages in germinating seeds of 12WH191 (P < 0.05; Additional file 1: Table S3). In contrast, other DEPs showed higher expression in germinating seeds of KenC8 compared to expression in 12WH191 seeds, including myrosinase-binding protein 2 (spot 51, 53 and 120), peptide methionine sulfoxide reductase B5 (spot 1668), epithiospecifier protein (spot 753 and 754), iron/manganese superoxide dismutase family protein (spot 1207), superoxide dismutase [Mn] (spot 1217), thioredoxin-dependent peroxidase 1 (spot 1319) and 1-cys peroxiredoxin PER1 (spot 1472) (*P* < 0.05; Additional file 1: Table S3). These results indicated that different stress- and defense-related proteins were activated or inhibited in response to internal and external stress and stimulation during seed germination, and that there might be significant differences in the occurrence and establishment of defense systems in the process of germination.

Thirty-two DEPs were involved in protein synthesis, folding, transport, modification and degradation, of which 21 protein spots were highly expressed in 12WH191 (Additional file 1: Table S3). For example, aspartic proteinase A1 (spot 980, 992 and 1655), protein disulfide isomerase-like 1–2 (spot 428, 432 and 436), elongation factor EF-2-like protein LOS1 (spot 97), heat shock cognate protein 70–1 (spot 292), heat shock protein 81–2 (spot 211), heat shock protein 70–4 (spot 286), luminal-binding protein 2 (spot 267), protein phosphatase 2A subunit A2 (spot 332), chaperonin-60 alpha (spot 407), TCP-1/cpn60 chaperonin family protein (spot 411), translational initiation factor 4A-1 (spot 633), etc., (Additional file 1:Table S3) were all highly expressed in 12WH191. In addition, 24 and 12 DEPs that are related to energy metabolism as well as sugar and carbohydrate metabolism involving tricarboxylic acid (TCA) cycling, glycolysis, gluconeogenesis, adenosine triphosphate (ATP) synthesis, glycerol and galactose metabolism were identified. In addition, most of these DEPs were also expressed significantly higher in 12WH191 compared to expression in KenC8 (P < 0.05), such as malate dehydrogenase and malate dehydrogenase 1 (spot 808, 816, 892 and 1094) and dihydrolipoyl dehydrogenase 1 (spot 438), which were highly expressed in high oil seedlings at several stages of germination (Additional file 1: Tables S3 and S5). It was shown that energy metabolism and carbohydrate metabolic activity might be significantly higher in 12WH191 than in KenC8, especially in the late stage of germination. In addition, there were 13 DEPs involved in amino acid metabolism, such as glutathione S-transferase (spot 1202, 1204, 1467 and 1709) and mercaptopyruvate sulfurtransferase 1 (spot 867), which were highly expressed in germinated 12WH191seeds.

Interestingly, we found that five DEPs (spot 125, 153, 370, 606 and 1108) were involved in signal response and conduction, and besides thioglucoside glucohydrolase 1 (spot 370), other DEPs were significantly higher in germinated seeds of 12WH191 than of KenC8 and reached their peak at 36 or 48 HAI (Additional file 1: Tables S3 and S5). Mature proteins in seeds and LEAs were also been identified; these proteins are involved in seed maturation and were highly expressed during seed development and maturation. The present results demonstrated that the expression level of these proteins was mostly reduced during seed germination, but the expression level of two LEAs (spot 192 and 506, late embryogenesis abundant protein) fluctuated during germination and post-germination seedling growth and were highly expressed after 36 HAI.

### Expression pattern analysis of the DEPs

**Fig. 5:**
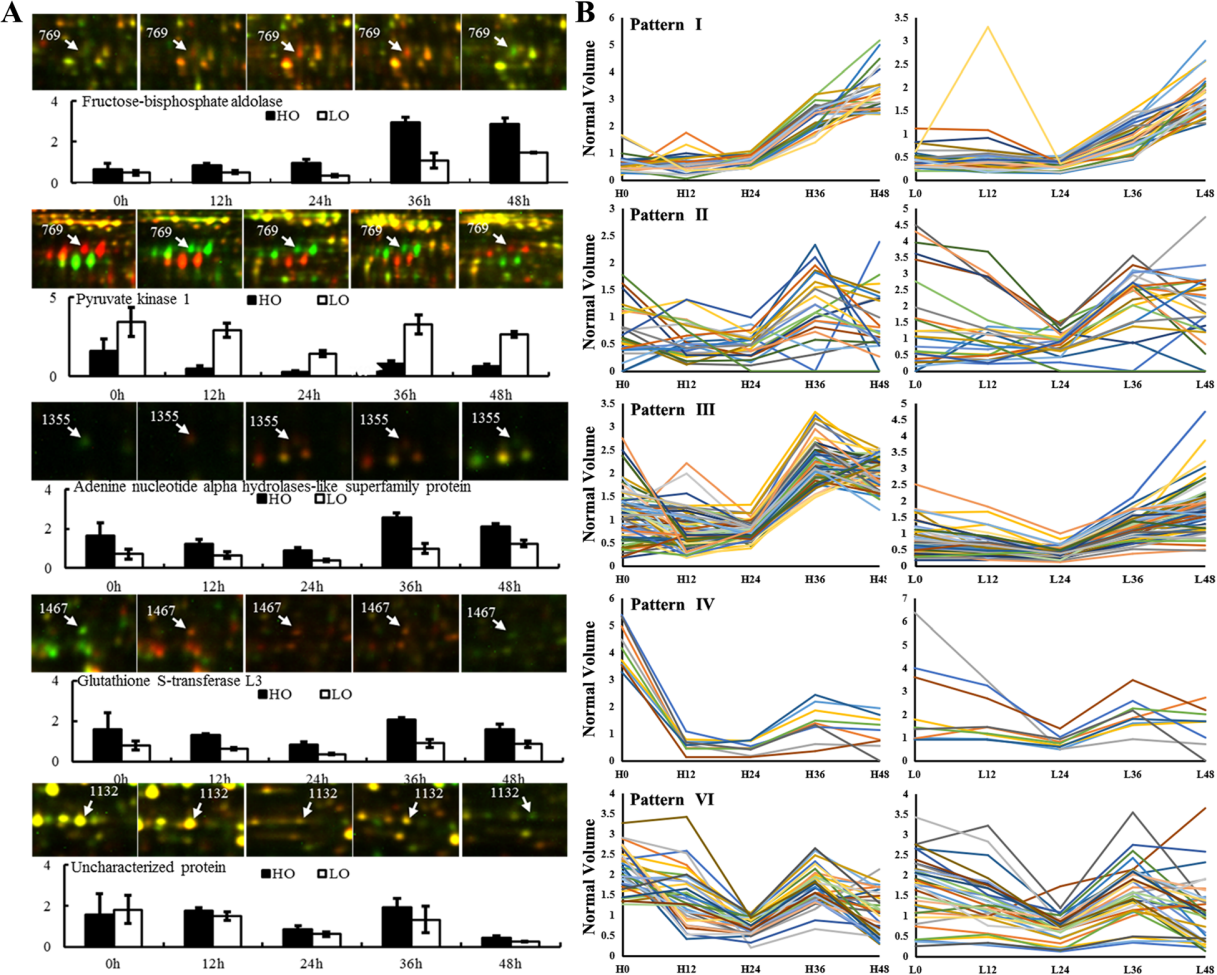
The K-mean cluster results of the DEPs. The DEPs were clustered according their relative expression changes by using SPSS K-mean clustering module. **a**, the representative protein spots. **b**, the K-means clustering results of the DEPs identified in the germination process of 12WH191 and KenC8

To fully understand the expression of these DEPs during germination, 165 DEPs were classified according to their expression changes in the germination process using SPSS 20 software. These DEPs could be divided into six groups (Patterns I-VI; Additional file 1: Table S5; Fig. 5b). Pattern V contained only one protein, aldolase-type TIM barrel family protein (spot 991); these DEP was highly expressed in dry seeds and then decreased rapidly and maintained a low expression level during subsequent germination and seedling growth (Additional file 1: Table 3 and S5). Patterns I (27) and III (70) represent the DEPs that slightly changed at the early stage of germination, followed by a significant increase in germination and seedling growth. Pattern I peaked at 48 HAI, and the expression peak of Pattern III was at 36 HAI. For example, in the seeds with high oil content, four DEPs that involved amino acid metabolism (spot 1202, 1204, 1467 and 1709, glutamate-cysteine ligase, 1-aminocyclopropane-1-carboxylate oxidase 2, mercaptopyruvate sulfurtransferase 1 and glutathione S-transferase F3) were decreased to the lowest expression level at 12 HAI and then increased rapidly from 24 HAI. However, the expression changes of DEPs in KenC8 lagged behind 12WH191; most expression fell into the lowest point at 24 HAI, and then began to rise rapidly. The same expression pattern was observed in the other DEPs between the two groups (Additional file 1: Table S5). Protein metabolism, energy metabolism, metabolism of sugar and carbohydrates, and stress- and defense-related proteins are the major protein species in the two clusters, indicating that substance and energy metabolism, new protein synthesis, modification and transport, and the construction of a defense system were activated during seed germination. In addition, four signal-related proteins (myrosinase 1, guanosine nucleotide diphosphate dissociation inhibitor 1,14–3-3-like protein GF14 kappa and protein low-temperature-induced 65) also appeared in Pattern III. Pattern II (24) represented the DEPs that had fluctuated expression during germination and seedling growth. In this cluster, protein metabolism, defense/disease and storage proteins were three major protein categories. The expression of 12S seed storage protein CRA1 (spot 1286, 1296, 1426 and 1539), cruciferin 2 (spot 1638) and cupin domain-containing protein (spot 249) gradually increased in early germination and then rapidly decreased at a different stage of germination (Additional file 1: Table S5). Both Pattern IV and Pattern VI indicate DEPs that decreased with germination but fluctuated during post-germination seedling growth, suggesting that these DEPs might play a more important role in the seedling growth stage. For example, some seed maturation proteins (spot 1012, 975, 988, 1132, 1431, 1476 and 528), cell growth and division-related proteins (spot 128 and 1357) (Additional file 1: Table S5) followed this pattern. At the same time, the expression patterns of these DEPs were significantly different between 12WH191 and KenC8 during seed germination and post-germination seedling growth, and some protein spots even showed the opposite expression pattern (Fig. 5, Additional file 1: Table S5).

**Fig. 6:**
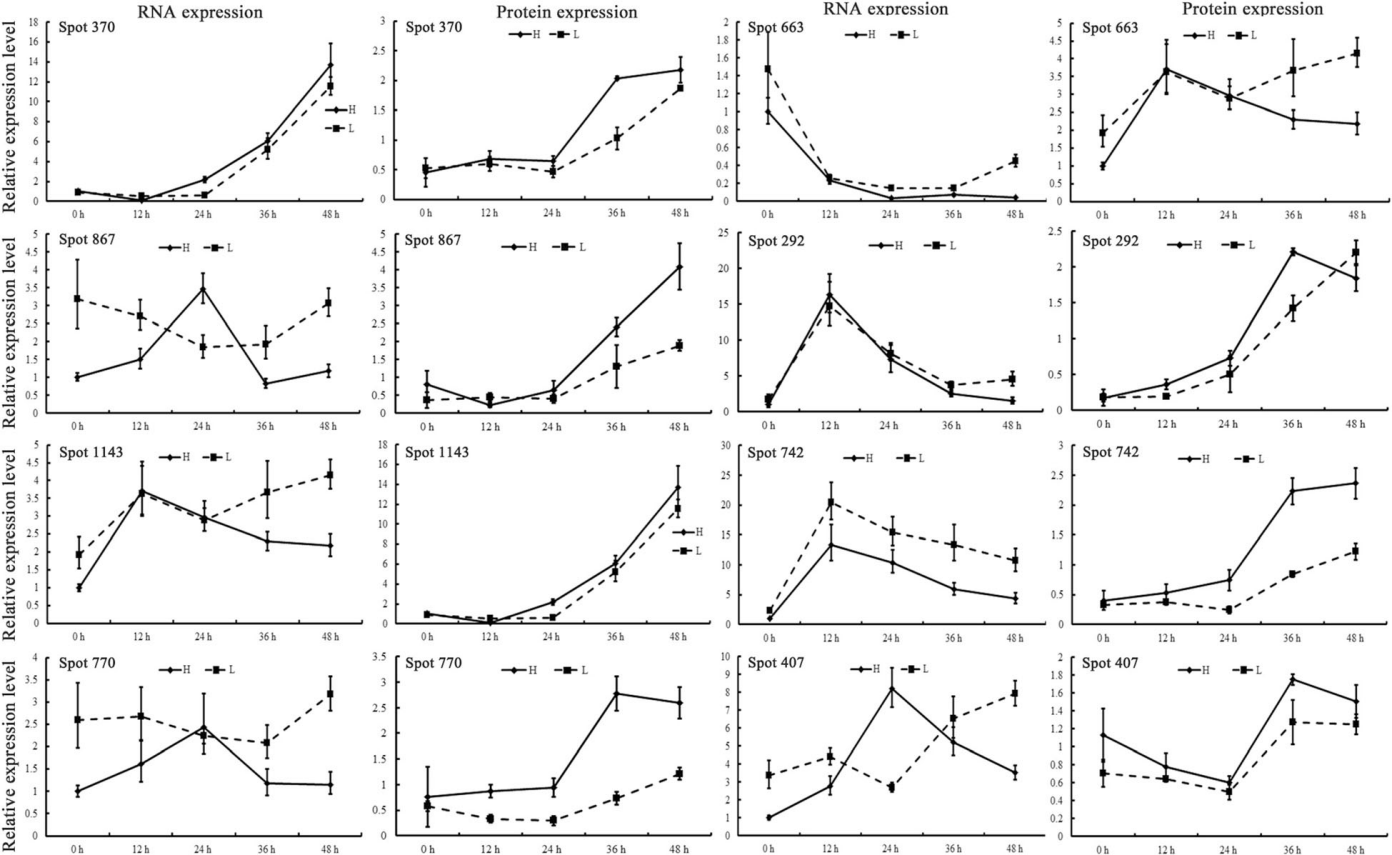
qRT-PCR analysis for DEPs. The transcriptional changes of 8 representative DEPs were analyzed by qRT-PCR and compared with corresponded DEPs. Spot 370: *AT5G26000*, thioglucoside glucohydrolase 1; Spot 867: *AT1G79230*, mercaptopyruvate sulfurtransferase 1; Spot 1143: *AT3G55440*, triosephosphate isomerase; Spot 770: *AT1G79550*, phosphoglycerate kinase; Spot 663: *AT1G77120*, alcohol dehydrogenase 1; Spot 292: *AT5G02500*, heat shock cognate protein 70–1; Spot 742: *AT4G33680*, LL-diaminopimelate aminotransferase; Spot 407: *AT2G28000*, chaperonin-60 alpha

Next, the gene expression of some DEPs was verified in germinating seeds with high and low oil content by qRT-PCR (Fig. 6). Although most of the genes and the corresponding DEPs showed similar changes in expression, some inconsistencies in gene expression and protein expression could still be observed, indicating that the differences in these protein levels might be caused by the regulation of transcription, translation, and post-transcriptional modifications.

### Interaction analysis of DEPs and comparison of metabolic pathways in germinating seeds with high and low oil content

**Fig. 7:**
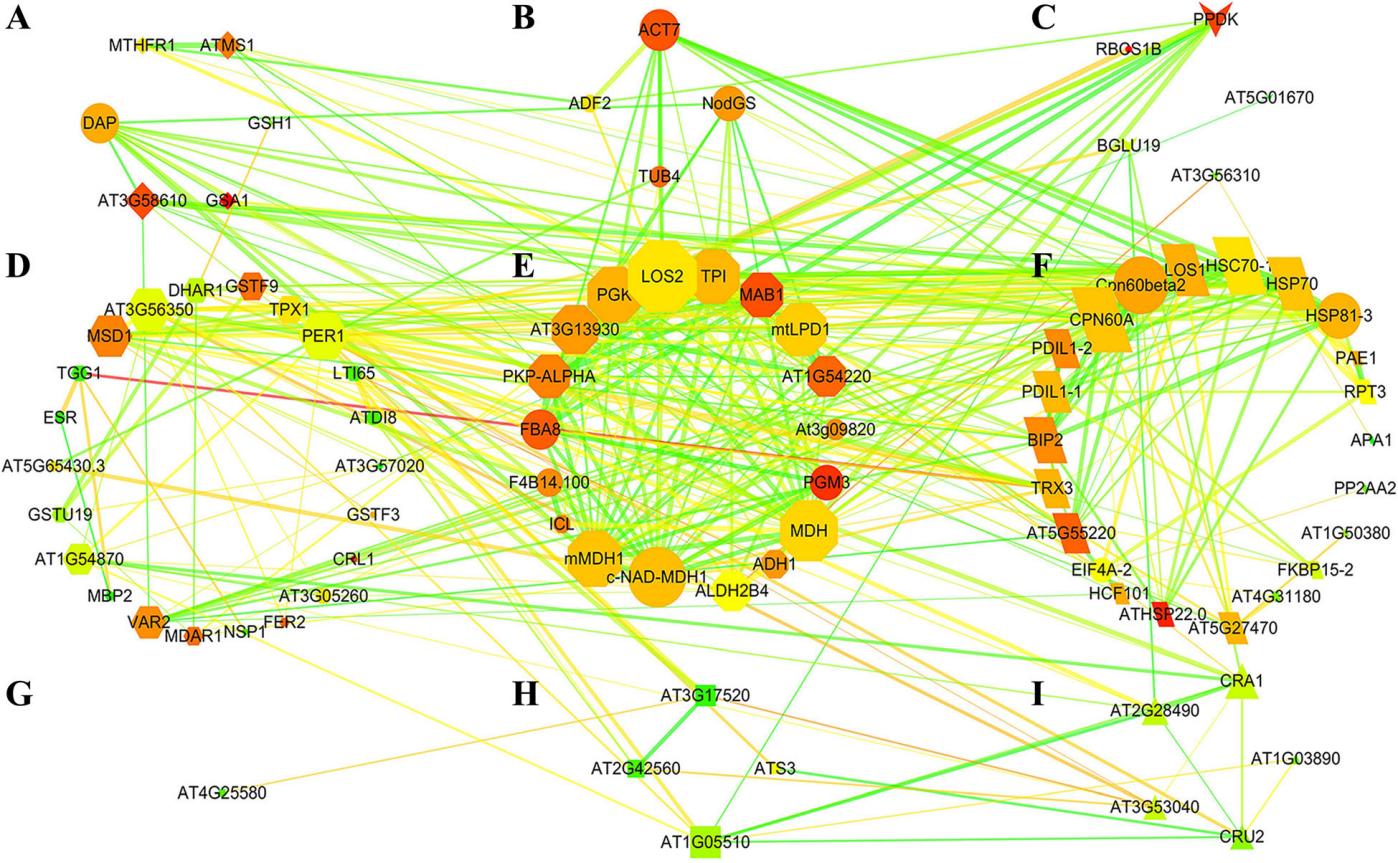
Network of DEPs identified in germinating *B. napus* seeds with high and low oil content. The associations between these 165 DEPs were analyzed by STRING 10.0, and then the protein association network was optimized with Cytoscape 3.2. A total 87 nodes and 400 lines are obvious in the network. The nodes represent DEPs and the lines represent protein-protein associations. The associations are meant to be specific and meaningful, i.e. proteins jointly contribute to a shared function and this does not necessarily mean they are physically binding each other. The thickness of line indicates the strength of data support for the associations among DEPs. These nodes were grouped into 10 subgroups according to their function. **a**, amino acid metabolism; **b**, cell growth/division; **c**, sugar metabolism; **d**, defense/disease; **e**, energy metabolism; **f**, protein metabolism; **g**, unknown function; **h**, seed maturation; **i**, storage proteins

The interaction between these 165 DEPs was analyzed by STRING 10.0, and then the protein interaction network was optimized with Cytoscape 3.2 (Fig. 7). The entire network contained 87 nodes and 400 lines. These nodes were grouped into 10 subgroups according to their function, including amino acid metabolism-related proteins, cell growth/division-related proteins, glucose metabolism-related proteins, defense/disease-related proteins, energy metabolism-related proteins, protein metabolism-related proteins, signal-related proteins, seed maturation-related proteins, storage proteins and protein of unknown function (Fig. 7, Additional file 1: Table S4). The proteins in these subgroups not only interacted with each other but were also closely related to the proteins in the other subgroups, forming a complex protein interaction network. For example, 18 proteins in subgroup E that involved in energy metabolism were closely linked to each other to form a complex network (Fig. 7) and were also closely related with other subgroups, such as protein metabolism-related proteins (Fig. 7f), amino acid metabolism-related proteins (Fig. 7a), cell growth/division-related proteins (Fig. 7b), and glucose metabolism-related proteins (Fig. 7c). It was shown that the energy metabolism-related process plays a core role in the complex physiological and biochemical metabolism of plants.

**Fig. 8:**
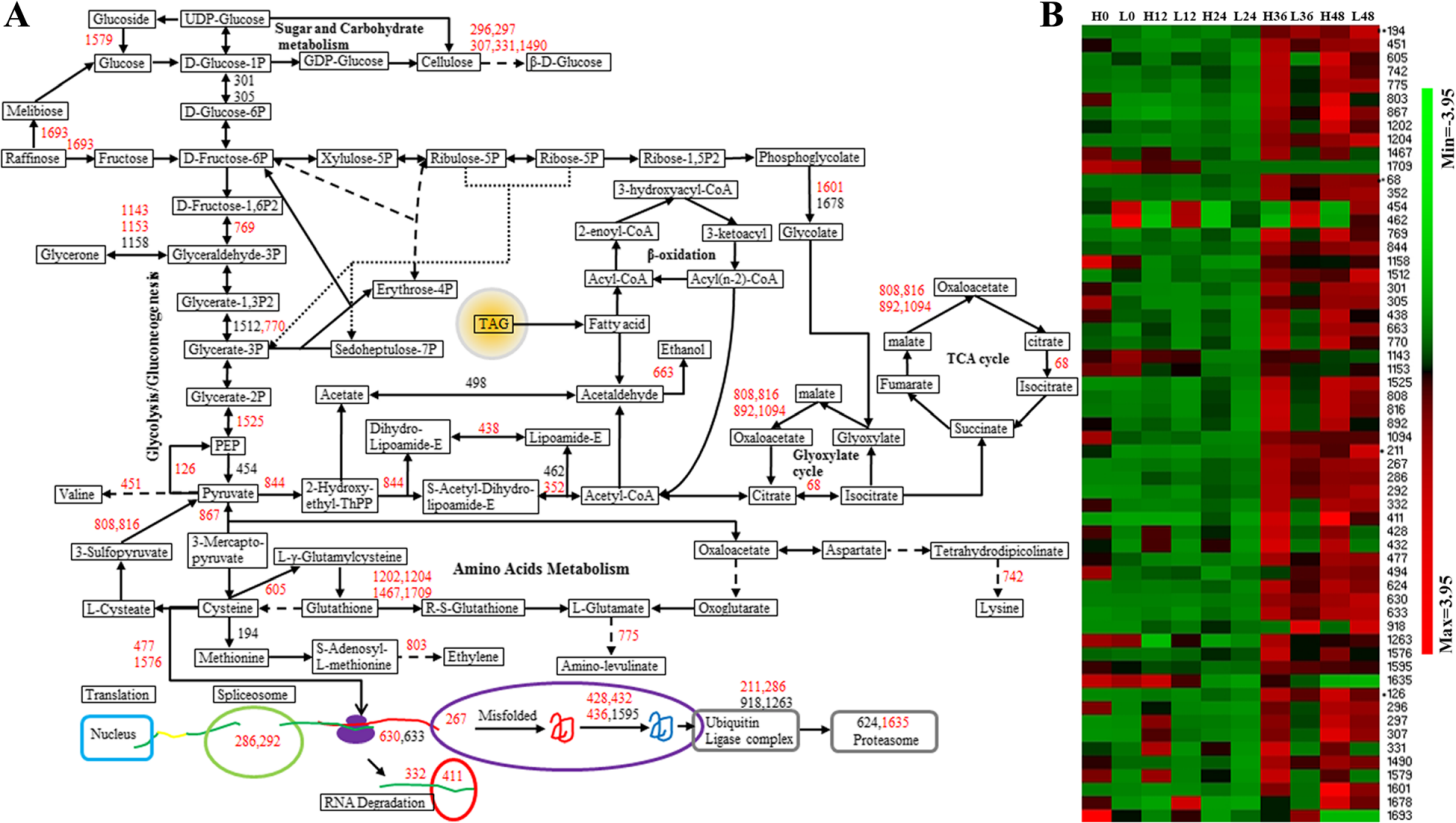
Potential metabolic pathways of DEPs during germination in *B. napus* seeds with high and low oil content. The potential metabolic pathways of DEPs were constructed based on searching the KEGG database and the interaction analysis of DEPs. Fifty-nine DEPs were localized in these potential metabolic pathways (**a**), and the relative expression level of these DEPs was also shown by heatmap (**b**). The red font indicates a protein spot that is highly expressed in germinated seeds with high oil content, and the black font indicates a protein spot that is highly expressed in germination seeds with low oil content

Based on searching the KEGG database (http://www.kegg.jp/) and the interaction analysis of DEPs, a metabolic pathway network of DEPs was constructed (Fig. 8). The network mainly contains the pathways of protein and amino acid metabolism, sugar and carbohydrate metabolism, glycolysis/gluconeogenesis, the glyoxylate cycle, TCA and so on. A total of 59 DEPs were localized in these metabolic pathways, and the expression patterns during the germination of these DEPs are also shown by heatmap (Fig. 8b). It was shown that protein and amino acid metabolism, sugar and carbohydrate metabolism, glycolysis, gluconeogenesis, glyoxylate cycle and TCA cycle were obviously more active in high oil-containing cultivar.

### QTL mapping and identification of important candidate genes that correspond to the DEPs

**Fig. 9:**
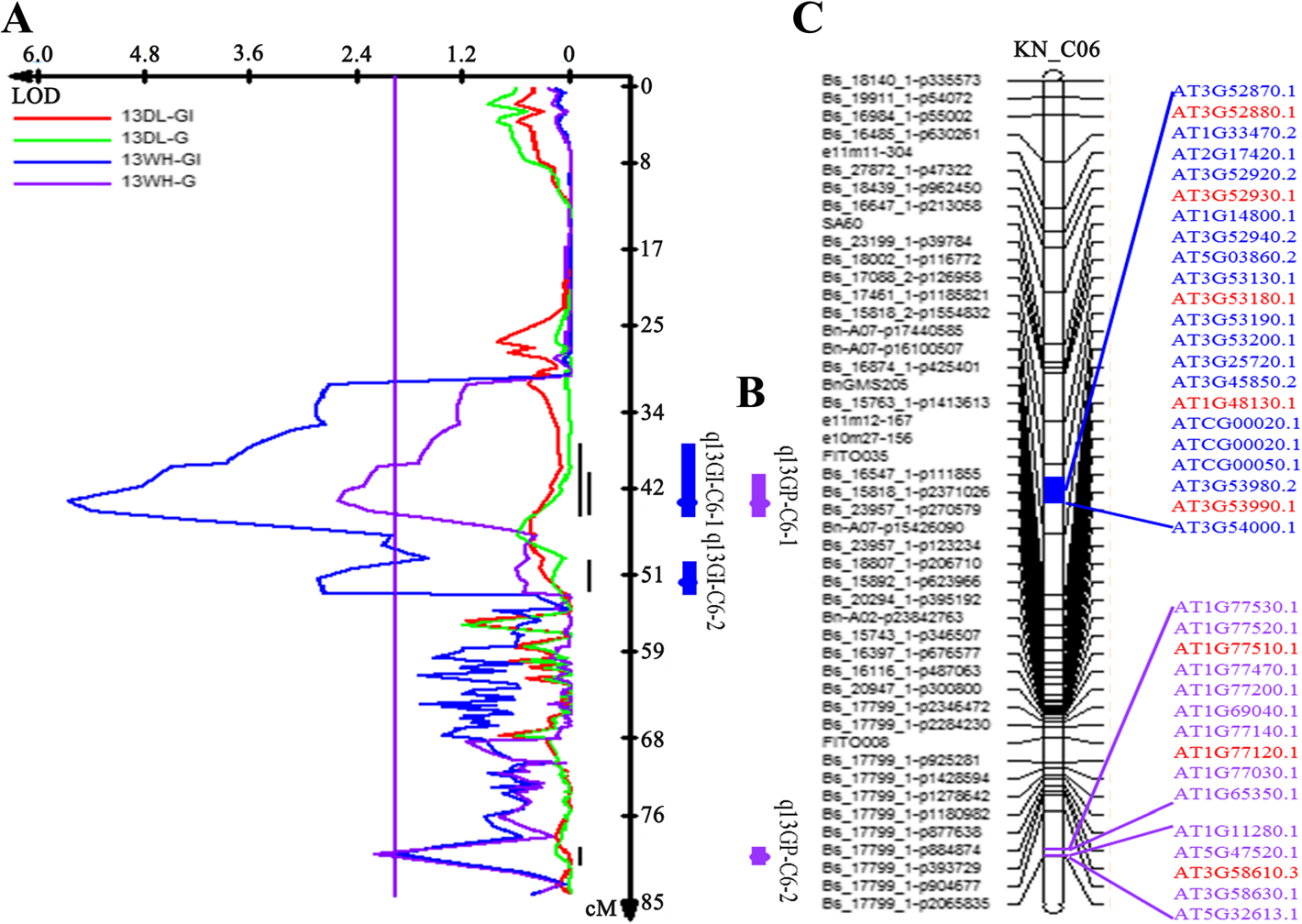
Co-localized QTLs for GP and GI on chromosome C6 and the annotation of candidate genes corresponding to the DEPs based on the Arabidopsis homologues. (**a**) QTL scanning results from seeds of the KNDH population that were harvested in two environments. Curves of different colors represent QTLs scanned from seeds of different environments. (**b**) The positions of the QTLs (including peak and confidence interval) identified on chromosome C6. (**c**) C6 linkage map of the KNDH population and the annotation of homologs. The left characters represent the molecular markers that exist on chromosome C6, the right characters represent the homologous genes that are located in the QTL confidence interval, and the red characters represent the homologous genes that correspond to the DEPs

The phenotypic and genetic association for the seed GI and germination potential (GP) were analyzed by QTL mapping. A total of 27 QTLs were identified based on the germination data of the seeds harvested from Wuhan and Dali in the year 2014, of which 15 QTLs were significant for GI with a PV of 3.47–12.90% and 12 of them showed significance for GP with a PV of 3.35–8.89% (Additional file 1: Table S6). Some QTLs controlled GI and GP at the same time, for example, the QTL *q13GI-C6–1* with a positive additive effect of 17.0624 and the QTL *q13GP-C6–1* with positive additive effect of 6.5475 (Fig. 9 a and b; Additional file 1: Table S6). In addition, *q13GI-C1–2* and *q13GI-C7–1* for GI were co-localized with *q13GP-C1* and *q13GP-C7–2* for GP with an additive effect of the same direction, respectively (Additional file 1: Table S6).

The DEPs were first screened as previously reported in the germinating *B. napus* seed [34]. Then, the genes corresponding to the DEPs that were obtained by proteomic analysis were used for alignment with the QTL intervals for GI and GP to verify whether the DEPs between the HO and LO cultivars were underlying the variation in seed germination vigor. The results revealed 31 unique genes that were also observed previously, and 13 genes of which were within the confidence intervals (CIs) of QTLs for GI and/or GP that were detected in the KNDH population (Additional file 1: Tables S7 and S8). *BnaC01g43710D (AT3G21380*, Mannose-binding lectin superfamily protein) was located in the CI of *q13GI-C1–1*. *BnaC06g19960D* (*AT1G79230*, mercapto-pyruvate sulfurtransferase 1), *BnaC06g20420D* (*AT1G78380*, glutathione S-transferase TAU 19), BnaA07g20290D (*AT1G79550*, phosphoglycerate kinase) and *BnaA07g20210D* (*AT1G79690*, nudix hydrolase 3) were located within the CI of *q13GI-C6–2*. *BnaA06g16950D*, which is homologous to *AT3G48000* and encodes aldehyde dehydrogenase 2B4, was identified underlying *q13GP-A3* and *q13GI-A3*. *BnaC04g42010D* (*AT5G02500*, heat shock cognate protein 70–1) was identified as underlying q13GP-C5 and q13GI-C5–4. *BnaC06g09330D* (*AT1G52030*, myrosinase-binding protein 2) and *BnaC03g70070D* (*AT3G13920*, translational initiation factor 4A-1) were identified in the CI of the major QTL, *q13GP-C6–1. BnaA07g33310D* (*AT1G77120*, alcohol dehydrogenase 1) and *BnaC06g38200D* (*AT1G77510*, protein disulfide isomerase-like 1–2) colocalized within *q13GP-C6–2* and *q13GI-C6–2*. *BnaC06g14990D* (*AT3G53990,* Adenine nucleotide alpha hydrolases-like superfamily protein) and *BnaC06g14670D* (*AT1G48130,* 1-Cys peroxiredoxin PER1) were located in the CI of *q13GI-C6–1* and *q13GP-C6–1* (Fig. 9; Additional file 1: Tables S7 and S8). The 13 genes that corresponded to the DEPs were identified within QTL CIs for GI and/or GP, indicating that these DEPs might be more related to seed germination vigor.

**Fig. 10:**
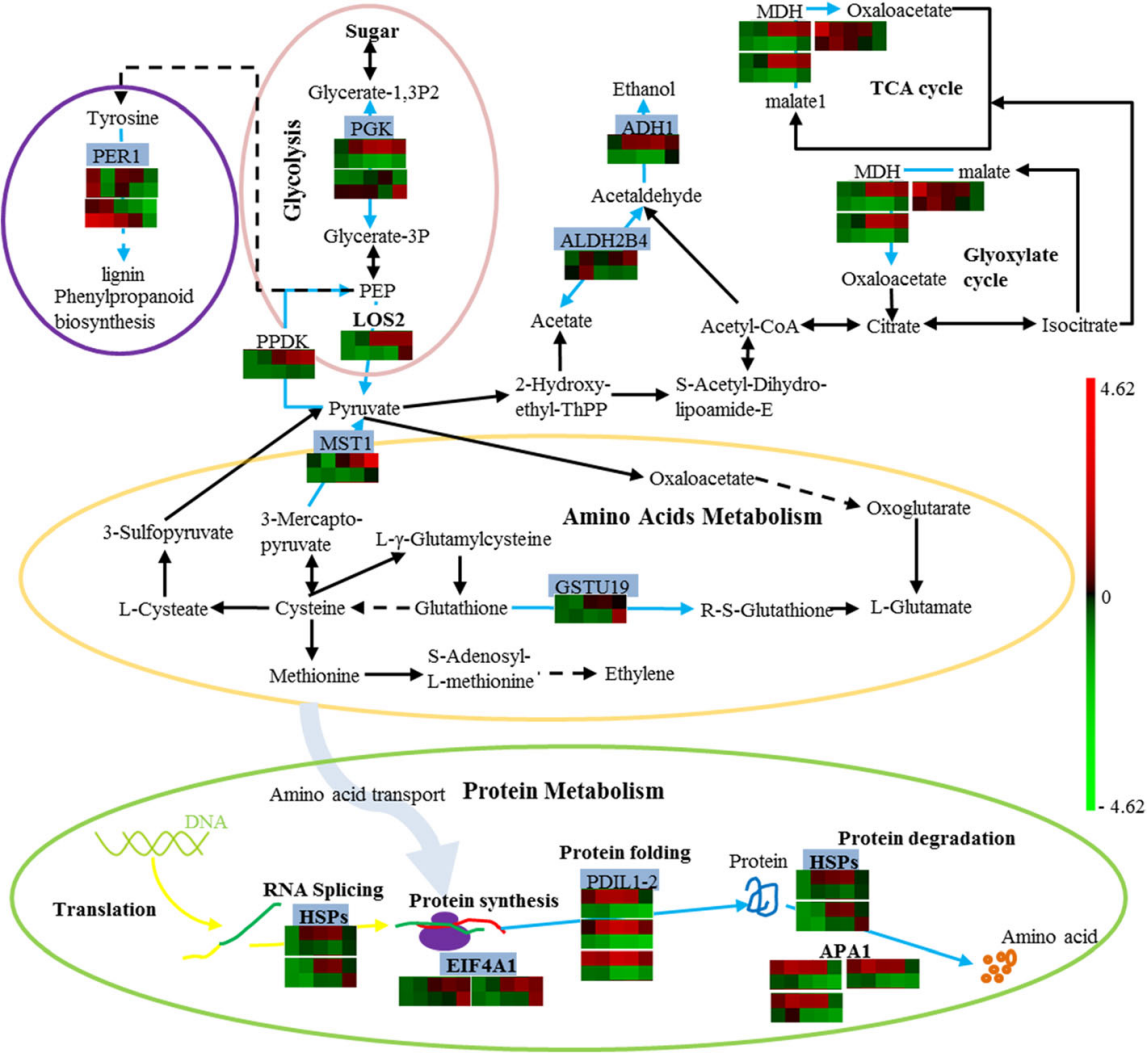
An integrated network of candidate gene-related metabolic pathways. The potential metabolic pathways network for candidate genes was constructed, and 15 of 31 candidate genes localized in the network. The relative expression changes of DEPs in the high and low oil-containing cultivars are shown by heatmap (upper and lower layer, respectively). The different colors indicate different relative protein expression level. The genes labeled with a blue background are the key candidate genes located within the CIs of QTL for GP and GI. LOS2: *AT2G36530*, bifunctional enolase 2/transcriptional activator; PPDK: *AT4G15530*, pyruvate, phosphate dikinase 1; PGK: *AT1G79550*, phosphoglycerate kinase; ADH1: *AT1G77120*, alcohol dehydrogenase 1; ALDHB4: *AT3G48000*, aldehyde dehydrogenase 2B4; MDH: *AT1G04410* and *AT1G53240*, malate dehydrogenase; PER1: *AT1G48130*, 1-cys peroxiredoxin PER1; MST1: *AT1G79230*, mercaptopyruvate sulfurtransferase 1; GSTU19: *AT1G78380*, glutathione S-transferase Tau 19; EIF4A1: *AT3G13920*, translational initiation factor 4A-1; HSPs: *AT3G12580* and *AT5G02500*, heat shock protein 70–4 and heat shock cognate protein 70–1; PDIL1–2: *AT1G77510*, protein disulfide isomerase-like 1–2; APA1: *AT1G11910*, aspartic proteinase A1

Then, the metabolic pathway network was also constructed, and 15 candidate genes were localized in the network (Fig. 10). These genes correlated with glycolysis, the TCA, phenylpropanoid biosynthesis, amino acid metabolism, RNA splicing, protein synthesis, folding and degradation, etc. *AT2G36530* (LOS2) encodes an enolase that catalyzes the reaction from 2-phosphoglycerate to phosphoenol-pyruvate in the glycolysis/gluconeogenesis pathway. *AT4G15530* (PPDK) encodes pyruvate orthophosphate dikinase and is involved in pyruvate metabolism. *AT1G79550* (PGK) encodes phosphoglycerate kinase and catalyzes the reaction of glycerate 1,3-diphosphate to glycerate 3-phosphate. *AT1G77120* (ADH1) catalyzes the reduction of acetaldehyde using NADH as a reductant. *AT3G48000* (ALDHB4) encodes a putative (NAD+) aldehyde dehydrogenase and can oxidize acetaldehyde. *AT1G04410* and *AT1G53240* (MDH) encode cytosolic and mitochrondrial malate dehydrogenase, respectively. Malate dehydrogenase might participate in the citrate cycle, cysteine and methionine metabolism and pyruvate metabolism. *AT1G48130* (PER1) is involved in phenylpropanoid biosynthesis and may protect seed tissue from reactive oxygen species during desiccation and early imbibition and/or is involved in the maintenance of/protection during dormancy. *AT1G79230* (MST1) encodes a sulfurtransferase, which catalyzes the transfer of sulfur from a donor to a thiophilic acceptor substrate and plays an important role in cysteine and methionine metabolism. *AT1G78380* (GSTU19) encodes a glutathione transferase, a member of the Tau GST gene family that functions as an s-transferase in glutathione metabolism. Five genes are located in the protein metabolism pathway. *AT3G13920* (EIF4A1) functions as translation initiation factor in protein synthesis. Heat shock proteins encoded by *AT3G12580* and *AT5G02500* are chaperone proteins which are associated with protein synthesis, modification and degradation. *AT1G77510* (PDIL1–2) encodes a protein disulfide isomerase-like protein and is involved in protein folding. APA1 (*AT1G11910*) encodes an aspartic proteinase and functions in protein catabolic processes. Moreover, ten of these genes could be found in the CIs of germination-related QTLs, which are involved in protein metabolism (3), energy metabolism (4), amino acid metabolism (2) and defense/disease (1) (Fig. 10). Most of these candidate genes are important metabolic genes, such as *AT1G79550* that encodes phosphoglycerate kinase and catalyzes the reaction of 1,3-diphosphoglycerate to 3-phosphoglycerate in glycolysis (Fig. 10).

## Discussion

### Oil content affects seed germination in *B. napus*

Seed vigor is the ability of seeds to quickly and precisely germinate and successfully build healthy seedlings, which is an important factor in determining crop yields [18, 20]. Seed vigor decreases with seed aging [18–20]. Some studies have shown that the seed vigor of *B. napus* is affected by artificial or natural aging treatments for seeds with different oil contents [9, 26]. The present results indicated that the average germination index for high oil content cultivars was slightly higher than that of cultivars with low oil content. The germination rate, germination initiation time and germination index of some high oil-containing cultivars were significantly higher than these measures in the low oil-containing cultivars. This result indicates that the seed germination vigor of *B. napus* might be affected by oil content.

### Abscisic acid and its signaling pathways may be related to the differences in seed vigor in *B. napus* with high and low oil content

ABA has been recognized as an important factor in determining plant seed germination [35]. During the transition from dormancy to germination, the susceptibility of seeds to ABA and indole-3-acetic acid (IAA) decreased [35]. A transcriptional analysis of the wheat seed response to ABA demonstrated that ABA inhibited the germination of wheat seeds by inhibiting chromosome assembly, cell wall modification, and the expression of gibberellin (GA)-metabolic activation-related genes [2, 36]. Morris et al. found that two genes (*BoLCVIG1* and *BoLCVIG2*) and a QTL (Reduced ABscisic Acid 1, RABA1) were associated with seed vigor in *Brassica oleracea*, which were demonstrated to be associated with ABA metabolism or the ABA sensitivity of seeds [28]. The germination rate of *B. oleracea* seeds might be determined by the combination of seed ABA content and seed ABA sensitivity [28]. Germination is regulated by the increasing growth potential of the radicle and the resistance of the testa and endosperm tissues covering the radicle [37]. Embryo elongation growth requires cell expansion in defined regions of the radicle and the lower hypocotyl, which is promoted by GA and inhibited by ABA [2, 38]. The resistance of the surrounding tissues could be reduced by enzymatic or physical weakening of their cell walls. Further, in *Lepidium sativum* and *Arabidopsis*, it has been shown that ABA delays endosperm rupture [39]. The liquid chromatograph mass spectrometer (LC-MS) analysis indicated that the content of ABA was higher in KenC8 during early germination (0–24 HAI). The endosperm rupture and the cell expansion in the radicle and lower hypocotyl may be delayed by the higher ABA content in *B. napus* seeds with low oil content. In addition, the present results showed that three DEPs, thioglucoside glucohydrolase 1 (spot 370) and protein low-temperature-induced 65 (spot 125 and 153), that associated with ABA-activated signaling pathways differed significantly between 12WH191 and KenC8 during germination. The protein low-temperature-induced 65 was highly expressed in 12WH191 throughout the germination process; otherwise, the expression of thioglucoside glucohydrolase 1 was significantly higher in the KenC8 initially and then it was more highly expressed in 12WH191 during the subsequent germination process. Although there are no reports on the role of thioglucoside glucohydrolase 1 in seed germination, previous studies have shown that it might be involved in ABA-related signaling pathways in leaf-guarding cells [40]. The protein low-temperature-induced 65 is induced by low temperature, drought, etc., and this induction is associated with ABA [41]. Therefore, the difference in seed vigor in *B. napus* cultivars may be related to seed ABA content and the ABA-related signaling pathways.

### Defense systems may contribute to the high germination vigor of seeds with high oil content

Seeds and seedlings are very sensitive to stress [42]. Therefore, the establishment of a defense system during seed germination may play an important role in ensuring normal seed germination. Many studies have emphasized the importance of cell defense mechanisms in the establishment and regulation of seed germination [43–45]. For example, low concentrations of salicylic acid (SA) can enhance the expression of some superoxide dismutase under salt stress. In addition, SA also enhances the re-induction of the late-maturing process in the early stages of germination, the glyoxylate cycle and methionine metabolism, as well as the mobilization of seed storage proteins. All of these SA-affected responses may help to improve the suitability of seeds for the environment and improve seed vigor [45]. The sulfur-containing amino acid metabolic pathway and the myrosinase-glucosinolate system play an important role in plant development and defense [46]. The sulfur amino acid metabolic pathway is a key determinant of whether the seed can initiate germination^2^, and inhibition of sulfur-containing amino acid metabolism can strongly delay seed germination and seedling growth [47–49]. Methionine is not merely a structural unit for protein synthesis; it also participates in the synthesis of S-adenosylmethionine and thus affects the synthesis of polyamines, ethylene and the vitamin biotin [50]. Glutathione and glutathione disulfide are essential for the reduction of various peroxides [51, 52]. Glutathione S-transferase plays a direct role in the response to external environmental stimuli, reduced oxidative damage, and damage from foreign substances [53, 54]. The present results revealed that 7 sulfur amino acid metabolism-related DEPs were highly expressed in high oil-containing seeds, and two corresponding genes (*AT1G79230* and *AT1G78380*) located within the CIs of QTLs for GP and GI were also identified. This suggests that higher sulfur-containing amino acid metabolic activity might help to improve seed suitability for the environment and seed vigor. The DEPs that correlated with the myrosinase-glucosinolate system were also identified in the germinating high and low oil-containing seeds; the myrosinase-binding protein 2 gene corresponding to the DEPs were located within the QTLs for GI and GP. There were three myrosinase-binding protein 2 spots (spot 59, 1516 and 1588) that were highly expressed in the seeds with high oil content, but the other three (spot 51, 53 and 120) were highly expressed in the seeds with low oil content, which indicated a complex relationship between the myrosinase-glucosinolate system and germination vigor.

### Seeds with higher oil content have more active substance and energy metabolism during germination

LEA and HSPs play an important role in maintaining seed vigor [25]. It was revealed that maize seeds with high vigor synthesized more heat shock proteins during germination, and five HSPs with molecular weights of 73 kD, 65 kD, 62 kD, 54 kD and 18 kD were shown to differ quantitatively between high and low vigor maize seeds. A special HSP with a molecular weight of 56 kD was only present in seeds with high vigor; thus, it might be used as an indicator of seed vigor [55]. Similarly, the heterogenously expressed HSP gene of lotus (*NnHSP17.5*; *Nelumbo nucifera Gaertn.*) in the *A. thaliana* could significantly enhance the germination potential of transgenic *Arabidopsis* seeds and improved the thermal tolerance of seedlings [56]. Consistent with this result, the high expression of LEA (spot 192, 506, and 528) proteins and HSPs (spot 211, 286 and 292) was identified in the proteomic experiments in the seeds with high oil content and was also detected in the CIs of the QTLs for GP and GI*.* Moreover, many important proteins related to protein synthesis, folding and modification were also highly expressed in the high oil-containing seeds, such as protein disulfide isomerase (spot 428, 432 and 436), chaperonin-60 (spot407 and 411), transcriptional initiation factor 4A-1 (spot 630) and tRNA synthetase (spot 477 and 1576). *AT1G77510* and *AT3G13920* encode protein disulfide isomerase and transcriptional initiation factor 4A-1, respectively, and were identified as located within the CIs of the QTLs for GP and GI. These DEPs might play an important role in ensuring the normal and rapid expression of the proteome during germination [57–59]. Protein synthesis is an absolute requirement for seed germination because the inhibition of protein translation can induce a complete block of *Arabidopsis* seed germination, and an apparent correlation between the protein translational ability and seed vigor has been identified [25, 60, 61]. A more active protein metabolism may be correlated with high seed vigor in *B. napus* seeds with high oil content.

In addition, the mobilization of seed storage reserves is one of the key mechanisms for maintaining seed vigor [25]. The glycolytic pathway is necessary for the mobilization of storage reserves during seed germination and is the main source of energy and material required for seed germination and seedling growth. Therefore, the glycolysis process is closely related to the rate of seed germination and seedling formation [2, 6, 62]. In the artificial aging of *B. napus* seeds, some metabolic-related proteins are inhibited by aging, which is thought to be associated with the reduction of seed germination vigor [27]. By comparing the differences in proteomics during the germination of high and low oil-containing seeds, we found that 10 amino acid metabolism-related proteins, 11 sugar and carbohydrate metabolism-related proteins, 15 proteins correlated with glycolysis/gluconeogenesis, TCA cycle and pyruvate metabolism and other energy metabolism processes were highly expressed in the high oil-containing seeds. Some genes corresponding to these DEPs were found to be located in the QTL confidence intervals for GI and GP by the QTL alignment analysis, such as *AT1G79550*, *AT1G77120* and *AT3G48000*, which encode phosphoglycerate kinase, alcohol dehydrogenase 1 and aldehyde dehydrogenase 2B4, respectively. Surprisingly, the total oil content in the dry matter increased significantly at 24 HAI in seeds with both high and low oil content and peaked at 48 and 36 HAI, respectively. A similar result was also observed in germinating jatropha seeds, but no relevant research focused on the mechanism underlying this phenomenon [63]. Embryo elongation growth requires cell expansion in defined regions of the radicle and lower hypocotyl during early seed germination, while cell division mainly occurs during post-germination seedling growth [2]. Large amounts of cell proliferation require the accumulation of phospholipids and may cause lipid metabolism to shift toward lipid regeneration. This requirement may be why the total oil content in dry matter increased significantly at 24 HAI. This increase in oil content was significantly higher in *B. napus* seeds with high oil content, suggesting that cell proliferation may be more exuberant in *B. napus* seeds with high oil content, which was consistent with seedling growth. These results showed that high oil-containing seeds have higher substance and energy metabolic activity during germination, which can accelerate storage substance mobilization, which provides the synthetic material and energy required for seed germination and seedling growth.

## Conclusions

Among the candidate genes we found were involved in the regulation of high and low oil seed germination vigor by the methods of QTL mapping and proteomics, only one gene (AT5G02500) was annotated as seed germination in the NCBI database. We speculated that there are two main reasons for this issue, (i) some DEPs have not been successfully detected, (ii) the difference in germination vigor between high and low oil content materials may not be attributed to the main genes involved in seed germination as reported previously. This might suggest that the regulatory mechanisms for germination vigor between high and low oil content *B. napus* seeds are more complex than we predicted. The present results not only emphasize the important role of protein synthesis, folding and degradation processes and the glycolysis process in the regulation of seed germination vigor but also suggest that sulfur amino acid metabolism and defense systems are also involved in the regulation of seed germination vigor. Some candidate genes are identified and further investigation of the functional analysis of these genes is needed in the future.

## Methods

### Plant materials

Twenty *B. napus* cultivars with different oil content were used (Additional file 1: Table S9). All the materials were stored in laboratory of Pro. Li Maoteng of huazhong university of science and technology. The seeds were imbibed in ultrapure water at 28 °C in the dark. The seeds/seedlings were collected for physiological indices, gene expression and proteomics analysis at 0, 12, 24, 36, 48, 60 and 72 HAI. Tissues were stored at − 80 °C until analysis.

### Seed germination index and transmission electron microscopy analysis

The number of newly germinated seeds was counted every 4 h, and then the seed germination index was calculated according to the described method [64].

Transmission electron microscopy (TEM) analysis was carried out according to the method described by Gan et al. [65]. Cotyledons of dry seeds and germinated seeds were collected and fixed with 2.5% glutaraldehyde for more than 24 h and then fixed with 1% osmium solution for 2 h. Next, the samples were dehydrated with gradient acetone (20, 50, 70, 90 and 100% × 3). Subsequently, the samples were infiltrated for 3 h in an acetone/epoxy resin mixture (acetone: epoxy, 2: 1) and epoxy resin and then infiltrated overnight using epoxy resin. The samples were finally embedded with epoxy resin and polymerized at 60 °C for 24 h. The samples were cut into 70 nm slices using a slicer (Leica MZ6, Germany), collected into a copper mesh and then stained with saturated uranium acetate and 0.4% lead citrate. The samples were rinsed with ultra-pure water 6 times, spaced with 15 s each time. Then, the sections were observed and imaged by a transmission electron microscope (JEM-1230, Japan).

### Oil content and fatty acid composition analysis

The change in oil content during seed germination and post-germination growth was detected following an improved Soxhlet extraction method [34]. Each sample was processed in three replicates. FA compositions were determined according to Browse et al. [66], using gas chromatography (GC-2010 Agilent, DEGS-diethyl glycol succinate column) with a flame-ionization detector. Relative FA compositions were calculated from five independent biological replicates.

### Total protein and sugar content analysis in germinating seeds

The Bradford method was used for total protein quantitation [67]. A standard solution (1 mg/ml γ-globulin) was used for the calibration curve, and 100 μl distilled water was used as the blank. Assay reagent (5 ml) was added into each tube and was well mixed by inversion or gentle vortex. The absorbance was measured at 595 nm. Protein samples (100 μl) were placed in centrifuge tubes and treated as described above. The total protein content of samples was calculated according to the standard curve.

Total sugar was analyzed with an improved method described by Wen et al. [68]. A glucose standard solution (100 μg/ml) was used for the calibration curve, and the standard curve was obtained by using the glucose content as the abscissa and the absorbance value as the vertical axis. The absorbance of the samples and the standard solution was analyzed at 620 nm. Then, the total sugar content of the sample was calculated according to the standard curve.

### Soluble sugar, pyruvate, acetyl-CoA and ABA analysis in germinating seeds

Soluble sugar was extracted with 1 ml deionized water in a boiling water bath for 10 min. The extracting solution was filtered into a 10 ml volumetric flask, and the volume was brought to 10 ml with deionized water [69]. Detection was performed according to the above method for three replicates.

The change of pyruvate content during *B. napus* seed germination was determined by the 2,4-dinitrophenylhydrazine method with a pyruvate content determination kit (Comin Biotechnology; CHN) [70]. Each sample was processed in three replicates.

The extraction and detection of acetyl-CoA was completed following a previously described method [71]. The final volume of the standards was 50 μl, and the concentrations of the standards were 60 pmol/L, 40 pmol/L, 20 pmol/L, 10 pmol/L and 5 pmol/L. Each concentration gradient was repeated twice. Ultrapure water (50 μl) was used as the blank control. The sample diluent (40 μl) was added to 10 μl of sample for testing. Absorbance was measured at 450 nm. The acetyl-CoA content was calculated according to the standard curve.

ABA was extracted from germinating seeds according to previous descriptions [72]. Then, ABA content was measured by following a derivatization approach as described previously, coupled with an API 4500 QTRAP LC/MS/MS system with a C18 column (WatersACQUITY UPLC HSS T3, 1.8 μm, 2.1 mm*100 mm) [73].

### Protein extraction and fluorescent dye labeling

The seeds/seedlings of 12WH191 and Ken C8 were collected for proteomics analysis at 0, 12, 24, 36 and 48 HAI. Protein was extracted according to the method described previously [34, 65]. Approximately 0.5 g of seeds/seedlings was homogenized to fine powder in liquid nitrogen and then transferred 0.1 g sample into a 2 ml Eppendorf tube with three replicates. Each sample was homogenized in 750 μl Tris saturated phenol (pH > 7.8) and 750 μl of homogenization buffer containing 0.1 M Tris-HCl (pH 7.5), 0.9 M sucrose, 10 mM ethylene diamine tetraacetic acid (EDTA), 0.4% (g/ml) dithiothreitol (DTT) in an ice bath for 30 min. The homogenate was centrifuged at 5000 g for 15 min at 4 °C. The supernatant was transferred to a new tube and mixed with 1.5 ml 0.1 M ammonium acetate-methanol solution in − 20 °C for overnight. The mixture was centrifuged at 5000 g for 10 min at 4 °C, and then the precipitation was washed twice with 0.1 M ammonium acetate-methanol solution and twice with acetone. The extracted protein was dried under room temperature for about 5 min and dissolved in a buffer solution containing 7 M urea, 2 M thiourea, and 4% 3-[(3-Cholanidopropyl)dimethylammonio]-1-propanesulfonate (CHAPS) (pH = 8.8) and then was frozen at − 20 °C for experiments. The protein concentration in the sample was tested using the 2D Quant kit (GE Healthcare, UK) kit according to the instructions. The concentration of all protein samples was adjusted to 5 μg/μl before it was labeled with DIGE fluorescent dyes [74]. For protein labeling, 50 pmol of CyDye(Cy2, Cy3 and Cy5) were mixed with 6 μl of protein sample and incubated on ice for at least 2 h in the dark. The labeling reaction was terminated by adding 1 μl of 10 mM lysine [74]. Three replicates of each sample were labeled with Cy3/Cy5 dyes as Additional file 1: Table S10. The internal reference were labeled with Cy2 by equilibrating all protein samples of equal volume [74]. Finally, mixes of the Cy2-, Cy3-, or Cy5-labeled protein were used for 2D-DIGE analysis.

### Gel electrophoresis, image scan, and data analysis

For 2D-DIGE analysis, the labeled proteins were mixed with 2D-DIGE buffer (7 M urea, 2 M thiourea, 4% CHAPS, 0.4% DTT, 0.5% IPG buffer) and separated with isoelectric focusing after being loaded on an immobilized pH gradient strip (IPG, pH 4–7, 24 cm; Amersham Biosciences, Uppsala, Sweden) [74]. The experimental conditions were followed as described by Gan et al. [65]. The IPG strips were rehydrated for 14 h, followed by holding at 100 V, 300 V, 600 V, 1000 V for 1 h at each step, then the voltage was raised to 10,000 V at linear mode and held until reaching a total value of 120,000 V-h. Then the IPG strips were equilibrated for 15 min each in an equilibration buffer [6 M urea, 0.375 M Tris−HCl (pH 8.8), 20% glycerol, and 2% DTT] containing 2% sodium dodecyl sulfate (SDS) and 2.5% iodacetamide, respectively. The Second dimension electrophoresis was performed on 12.5% SDS-polyacrylamide gel with an Ettan DALT six electrophoresis system (GE Healthcare, UK). The electrophoresis was performed at 50 V for 2 h and then at 110 V until the bromphenol blue front arrived to the bottom of the gel. A Typhoon 8600 scanner (GE Healthcare, UK) was used for image collection and these images were then analyzed by DeCyder 6.5 software (GE Healthcare, UK) according to Gu et al. [34] Spot detection was performed by the differential in-gel analysis (DIA) module by setting the estimated spots number as 3500. After removing the artifact spots by manual editing, the images were further analyzed by the biological variation analysis (BVA) module. Three biological replicates were used for statistical analysis of each treatment. Spots showing a ≥ 1.2-fold change in spot volume were considered as differentially expressed spots (T-Test, *P* < 0.05).

### Protein spot picking and identification

For spot picking, 1 mg protein samples were loaded onto IPG strips for isoelectric focusing, and then they were separated by 2D electrophoresis as described in the method above [65]. Gels were stained with coomassie brilliant bule R-250 (CBB R-250) and scanned by a UMAX Power Look 2100XL scanner (UMAX, Inc., Taipei, China) [65]. Then, the DEPs were selected by matching the spot-picking gel map and DIGE map, and then were manually excised from the gels for mass spectrometry (MS) and MS/MS analysis [65, 74–76].

The gel pieces were distained with 50 mM NH4HCO3 in 50% (*v*/v) methanol for 1 h at 40 °C twice. Then the protein in the gel piece was reduced with 10 mM EDTA, 10 mM DTT in 100 mM NH4HCO3 for 1 h at 60 °C and incubated with 10 mM EDTA, 40 mM iodoacetamide in 100 mM NH4HCO3 for 30 min at room temperature under the dark. The gel pieces were minced and lyophilized, then rehydrated in 25 mM NH4HCO3 with 10 ng of sequenced grade modified trypsin (Promega, Madison, WI) at 37 °C overnight. After digestion, the protein peptides were collected, and the gels were washed twice with 0.1% trifluoroacetic acid (TFA) in 50% acetonitrile to collect the remaining peptides. The peptides were desalted by ZipTipC 18 pipet tips (Millipore, Bedford, MA) and co-crystallized with 1 volume of saturated R-cyano-4-hydroxycinnamic acid in 50% (v/v) acetonitrile containing 1% TFA.

The desalted protein samples were subject to ABI 5800 MALDI-TOF/TOF Plus mass spectrometer (Applied Biosystems, USA), using a surveyor high performance liquid chromatography (HPLC) system. Data were acquired in a positive MS reflector using a CalMix5 standard to calibrate the instrument (ABI5800 Calibration Mixture). The mass spectrometry range of peptide mass fingerprinting (PMF) was set to 800–3500 Da and followed by 10 MS/MS scans on the 10 most intense ions from the MS spectrum. Both the MS and MS/MS data were integrated and processed by using the GPS Explorer V3.6 software (Applied Biosystems, USA) with default parameters. Based on combined MS and MS/MS spectra, proteins were successfully identified based on 95% or higher confidence interval of their scores in the MASCOT V2.3 search engine (Matrix Science Ltd., London, U.K.), using the following search parameters: NCBInr-*Brassica napus* database; trypsin as the digestion enzyme; one missed cleavage site; fixed modifications of Carbamidomethyl (C); partial modifications of Acetyl (Protein N-term), Deamidated (NQ), Dioxidation (W), Oxidation (M); 100 ppm for precursor ion tolerance and 0.5 Da for fragment ion Tolerance [34].

### Protein classification, hierarchical clustering analysis and interaction analysis of DEPs

DEPs were classified into different categories using NCBI (https://www.ncbi.nlm.nih.gov/), TAIR (http://www.arabidopsis.org/index.jsp) and KEGG (http://www.genome.jp/kegg/) databases. To visualize the expression characteristics of the DEPs, a hierarchical cluster was constructed using the K-means clustering approach in SPSS [77].

Interactions between DEPs were analyzed by STRING 10.0 (http://string-db.org). The locus tag of the *Arabidopsis* homologous corresponding to the DEPs and DEGs were used as inputs for STRING 10.0 to create the interaction network. The main interaction analysis parameters were as follows: The organism was set to *A. thaliana*; the interaction analysis was based on the default selection for all options, including text mining, experiment, database, coexpression, neighborhood, gene fusion and simultaneous occurrence; the interaction score was set to medium confidence (i.e., the interaction score is 0.4); the remaining parameters were selected as the default. In addition, the network was then displayed by Cytoscape 3.2.

### QTL mapping of seed germination index and germination potential

The high-density linkage genetic map used for QTL mapping of the seed germination index and germination potential was constructed based on the segregating double haploid (DH) population, named the KNDH population by Chao et al. [29]. The KNDH population was derived from a cross between the parental lines KenC-8 with low oil content and N53–2 with high oil content. The seeds of the KNDH population that were used for the germination index and germination potential analysis were collected from Wuhan of Hubei Province (a semi winter-type rapeseed growing area, coded WH) and Dali of Shannxi Province (a winter-type rapeseed growing area, coded DL) in 2014. The QTL analysis for the seed germination index and seed germination potential was performed as described by Chao et al. [29]. Putative QTLs was detected by Windows QTL Cartographer 2.5 under the CIM model. The scan walking speed was 2 cM, and the window size was 10 cM with five background cofactors. The LOD threshold (< 2.0) for the detection of QTLs was calculated by a 1000-permutation test based upon a 5% experiment-wise error rate. All of these QTLs were named by combining the microenvironment and the chromosome number, such as q13GI-C1–2. Gene alignment and candidate gene identification within QTL regions were performed according to the method that was described by Chao et al. [29].

### Gene expression analysis of candidate genes

An RNAprep Pure plant kit (TOYOBO, Japan) was used for the extraction of total ribonucleic acid (RNA). According to the user manual, the first chain was synthesized using the ReverTra Ace qPCR RT Master Mix with gDNA Remover (TOYOBO, Japan). The ABI 7900HT Fast Real-Time PCR System (Applied Biosystems, USA) was used for gene expression analysis with the SYBR premix EX TaqTM kit (TaKaRa, Japan). Relative gene expression was calculated according to Pfaffl’s method [78]. Each sample was completed in three replicates for calculating standard deviation (SD) errors to evaluate the intra-assay variation. *BnaActin 2.1* was used as the reference gene [79]. The primers that were used in this study are listed in Additional file 1: Table S11.

## Additional files

Additional file 1: **Table S1.** The result of ANOVA analysis for germination index and germination rate. **Table S2.** The peptide matches of DEPs. **Table S3.** The DEPs identified during germination in seeds with high and low oil content in *B. napus*. **Table S4.** Corresponding Arabidopsis homologous genes and STRING abbreviations of DEPs. Bolded font represents the genes that annotated as seed germination in NCBI database. Italic font represents the genes that annotated as seed specific in NCBI database. **Table S5.** Expression patterns of DEPs in germinating seeds with high and low oil content. H and L indicates that protein expression patterns of high and low oil-containing seeds, and the number indicates the hours after imbibition. **Table S6.** All of the QTLs for GP and GI detected in the present research. **Table S7.** The candidate genes identified by proteomic analysis. **Table S8.** The DEPs located within the QTLs for GP and GI. Black bold represent the gene that also founded in Table S7. **Table S9.** *B. napus* cultivars used in this research. **Table S10.** The setting of fluorescent dye labeling for protein samples. **Table S11.** The primers used in the present research. (XLSX 272 kb)

Additional file 2: Comparison of germination in *B. napus* with different oil content. A, the germination rates of seeds with different oil content. B, the germination index of seeds with different oil content. H1: 14QT083 (51.99%); H2: 14QT113 (52.84%); H3: 14QT101 (53.77%); H4: 14QT141 (54.34%); H5: 14QT090 (55.24%); L6: 14QT027 (41.93%); L7: 14QT023(43.21%); L8: 14QT077 (43.82%); L9 14QT044 (44.32%); L10: 14QT121 (44.55%). GI-H and GI-L represent the average germination index of the high oil and low oil groups, respectively. (TIF 1115 kb)

Additional file 3: Comparison of moisture, oil, protein and sugar of *B. napus* seeds between 12WH191 (H) and KenC8 (L) cultivars in the imbibition process. Values are the means of three biological replicates (SD). (TIF 2100 kb)

Additional file 4: The FA compositions of the crude oil from different stages of germination in 12WH191 (H) and KenC8 (L) seeds. (TIF 2271 kb)

## Abbreviations

2D-DIGE: Two-dimensional fluorescence difference in gel electrophoresis
ABA: Abscisic acid
ATP: Adenosine triphosphate
BVA: Biological variation analysis
CBB R-250: Coomassie brilliant bule R-250
CHAPS: 3-[(3-Cholanidopropyl)dimethylammonio]-1- propanesulfonate
CI: Confidence interval
DEPs: Differentially expressed proteins
DIA: Differential in-gel analysis
DTT: DL-Dithiothreitol
EDTA: Ethylene Diamine Tetraacetic Acid
FA: Fatty acid
GI: Germination index
GP: Germination potential
HAI: Hour after imbibition
HO: High oil content
HPLC: High performance liquid chromatography
HSP: Heat shock protein
IPG: Immobilized pH gradient
LEA: Late embryogenesis abundant protein
LO: Low oil content
MS: Mass spectrometry
PCR: Polymerase Chain Reaction
PMF: Peptide mass fingerprinting
PV: Phenotype variation
QTL: Quantitative trait locus
RNA: Ribonucleic acid
SA: Salicylic acid
SD: Standard deviation
SDS: Sodium dodecyl sulfate
TCA: Tricarboxylic acid cycle;
TEM: Transmission electron microscopy
TFA: Trifluoroacetic acid
