# Supplementary figures and images for: Integration of proteomic and genomic approaches to dissect seed germination vigor in Brassica napus seeds differing in oil content
Source: BMC Plant Biol. 2019 Jan 11;19:21. doi: 10.1186/s12870-018-1624-7 (PMC6329107; doi:10.1186/s12870-018-1624-7)

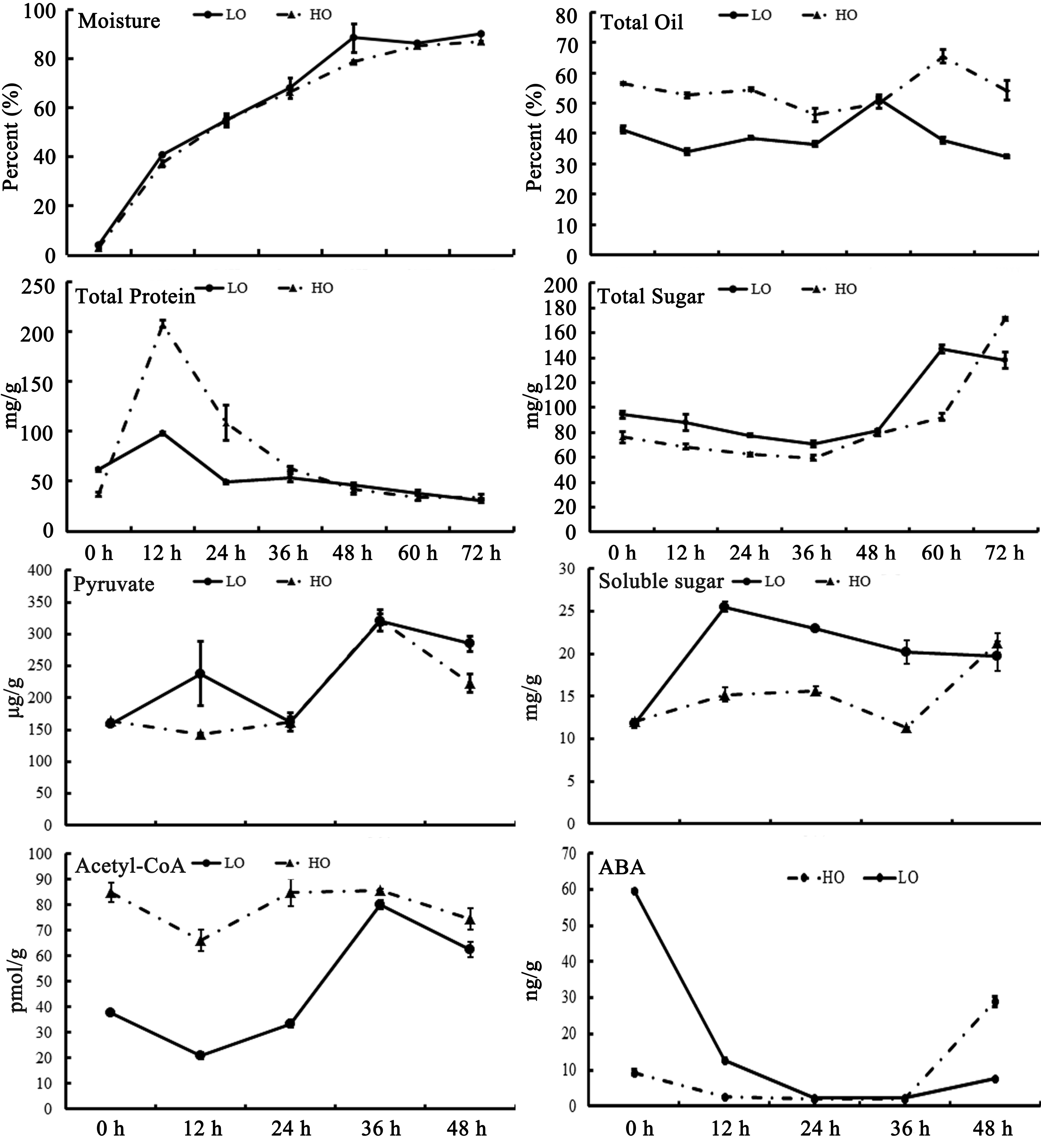

Supplement: Supplementary file 3 — Comparison of moisture, oil, protein and sugar of B. napus seeds between 12WH191 (H) and KenC8 (L) cultivars in the imbibition process. Values are the means of three biological replicates (SD). (TIF 2100 kb) [file 12870_2018_1624_MOESM3_ESM.tif]

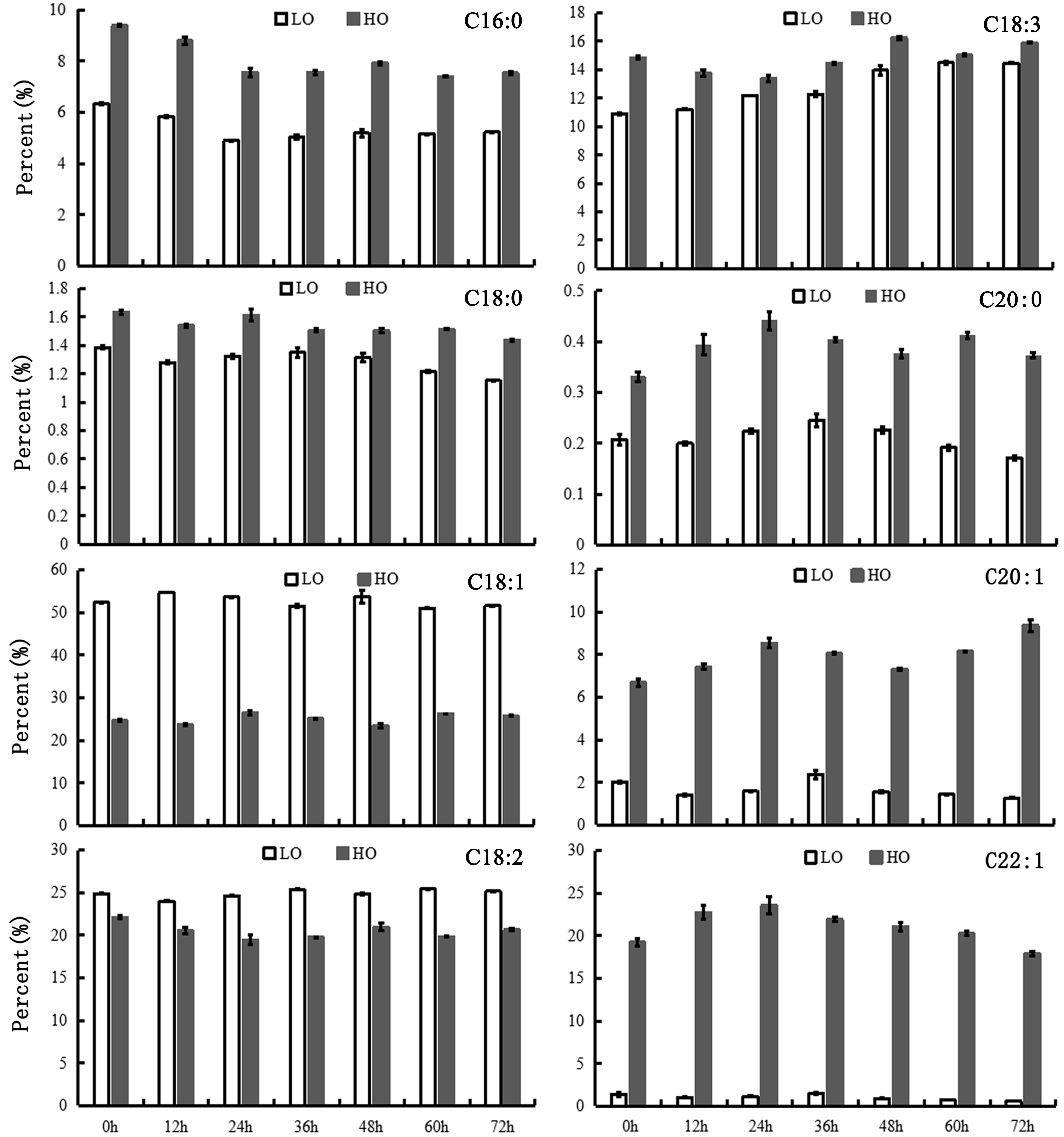

Supplement: Supplementary file 4 — The FA compositions of the crude oil from different stages of germination in 12WH191 (H) and KenC8 (L) seeds. (TIF 2271 kb) [file 12870_2018_1624_MOESM4_ESM.tif]
